# A systematic review of case reports and case series of zolpidem in the diagnosis and treatment of catatonia

**DOI:** 10.1177/20451253261487038

**Published:** 2026-09-25

**Authors:** Niraj Ahuja, Harry Hackett, Joseph Thorne, Stuart Watson, Richard Hamish McAllister-Williams

**Affiliations:** 1 5633 Regional Affective Disorders Service (RADS), Cumbria, Northumberland, Tyne and Wear NHS Foundation Trust, Newcastle Upon Tyne, UK; 2 Translational and Clinical Research Institute, 5994 Newcastle University, Newcastle Upon Tyne, UK; 3 Regional Affective Disorders Service (RADS), NIHR Newcastle Biomedical Research Centre (BRC), Newcastle Upon Tyne, UK

**Keywords:** catatonia, zolpidem, benzodiazepines, lorazepam, GABA-A receptor

## Abstract

**Background:**

Catatonia, commonly seen in psychiatric and medical practice, is often associated with increased morbidity and mortality. The neurotransmitter systems involved in catatonia include GABA, dopamine and glutamate. Benzodiazepines such as lorazepam, which act on GABA-A receptors, are used to confirm the diagnosis of catatonia, and are the mainstay of treatment. However, several patients with catatonia either don’t respond to lorazepam or its use is not suitable or possible (e.g. due to supply issues). Zolpidem is a positive allosteric modulator of GABA-A receptors. There are an increasing number of case reports and one systematic review of its use in the diagnosis and treatment of catatonia.

**Objectives:**

The objective of this systematic review is to explore the published evidence of zolpidem’s efficacy as a diagnostic test and for treatment for catatonia.

**Design, data sources and methods:**

We systematically reviewed evidence for zolpidem use in diagnosis and/or treatment of catatonia, using the PRISMA guidelines. We identified 44 case reports and case series, with 79 patients who met the inclusion criteria through searches of PubMed, Medline, EMBASE, CINAHL, ProQuest and PsycInfo.

**Results:**

67 out of 79 patients (84.8%) with catatonia showed a positive response to zolpidem, with remission of catatonic symptoms in 24.1% of patients. The high response rate was evident regardless of its use as a challenge test (85.7%) or treatment (86.1%), either used alone (85.7%), or with a benzodiazepine (81.3%). The mean dose of zolpidem was 14.7mg, with a median of 10mg, and a range of 5 to 45mg, with a rapid mean time to response of 30.2 minutes. A few patients responded better to zolpidem at a higher dose and/or when used over a longer duration though this was not always the case. The response rate was higher in women (88.6%) as compared to men (64.7%).

**Conclusions:**

Although the results from this systematic review generate optimism for the use of zolpidem in diagnosis and treatment of catatonia, these results should be used with caution as there are no randomised controlled trials of zolpidem compared to placebo and/or lorazepam.

## Plain language summary

This systematic review found 44 published case reports and case series which report 79 cases of zolpidem use in catatonia. 67 out of 79 patients (84.8%) with catatonia showed a positive response to zolpidem, with remission of symptoms in 24.1% of patients. Over 4 in 5 patients (86.1%) responded to zolpidem treatment. A similar proportion of patients (85.7%) responded when zolpidem was used as a diagnostic test. The positive response was similar whether zolpidem was used alone (85.7%) or with benzodiazepines (81.3%). Some patients respond better to zolpidem at a higher dose and/or use over longer duration, but this needs further study. This systematic review recommends further studies of zolpidem as a possible alternative to lorazepam in diagnosing and managing catatonic presentations before its use becomes common practice.

## Introduction

Catatonia is a relatively common^1^ clinical syndrome associated with a wide variety of psychiatric, medical and neurological disorders.^2–4^ Movement and speech are affected and there may also be autonomic and affective components.^5^ The pathophysiology of catatonia is poorly understood though most authors agree that the relevant brain areas include the cortico-striato-thalamo-cortical loop, supplementary motor area, prefrontal cortex, cingulate cortex and limbic system, and basal ganglia.^4,5^ There is hypothesised dysfunction in GABAergic, dopaminergic, glutamatergic and serotonergic function in catatonia.^6–7^

Early recognition can help ameliorate the significant morbidity and mortality associated with catatonia, however, it is still often under-detected and under-diagnosed, particularly with the excited and mixed forms of catatonia.^1^ Catatonia can lead to several complications including deep vein thrombosis, pulmonary embolism, aspiration pneumonia, decubitus ulcers, and death.^4^

Over the last four decades, benzodiazepines (e.g., lorazepam) have effectively replaced barbiturates^8,9^ as a diagnostic challenge test for catatonia. Lorazepam^10^ and diazepam^11^ are both non-selective positive allosteric modulators of GABA-A receptors and are effective in confirming the diagnosis of catatonia. The “lorazepam test” is widely used.^1^ Lorazepam is available in both oral and parenteral (IV and IM) forms and has a shorter half-life than diazepam. There is less robust case-report evidence for the use of other benzodiazepines such as clonazepam,^12^ midazolam,^13^ and oxazepam.^14^

Most recommendations^1,4,7^ for treatment of catatonia suggest benzodiazepines (usually lorazepam) as first line, with electroconvulsive therapy (ECT) as an alternative first or second line treatment.^4^ Some patients may not be suitable for ECT, and access to ECT can sometimes be challenging.^15^ Parenteral lorazepam, which has become a norm as a test for catatonia,^1^ is not available everywhere^16–20^ and some patients do not always respond to lorazepam.^6^ It is therefore useful to consider alternative options for assessing and treating catatonia.

Like lorazepam, z-drugs bind to the benzodiazepine site located at the interface between an α and γ2 subunit of the GABA-A receptor complex, with the identity of the α subunit as the primary determinant of a drug’s binding affinity and subsequent effects. The most common synaptic GABA-A receptor composition in the brain is α1β2γ2. Zolpidem, an imidazopyridine z-drug, has a rapid onset and is a commonly prescribed non-benzodiazepine hypnotic. It has been hypothesised to have utility in the diagnosis and treatment of catatonia, in part because it is a selective positive allosteric modulator of the GABA-A α1 sub-unit.^1,7,21^

This systematic review explores the published evidence testing efficacy of zolpidem as a diagnostic test and for treatment for catatonia.

## Material and methods

We followed the PRISMA (The Preferred Reporting Items for Systematic Reviews and Meta-analysis) statement guidelines^22^ for writing this systematic review (see Appendix 1 for the completed checklist). Though the protocol for this systematic review was not pre-registered on the PROSPERO database, a clear account of search strategy, criteria and technique for selection of studies, and data extraction strategies had been agreed a priori before progress with data analysis.

We used PubMed, Medline, EMBASE, CINAHL, ProQuest, and PsycInfo from their start dates to 31/05/2025, with search terms of (catatonia OR catatonic OR cataton*) AND zolpidem. N.A. completed de-duplications after two searches which were confirmed by H.H., with S.W. acting as arbitrator in case of any disagreement. We included all English-language articles published in a peer-reviewed journal where an abstract and/or a full text article was available. Articles published in other languages where an English language translation was available were included. We excluded articles with a lack of requisite information, those that reviewed the literature without a case report, reporting catatonia only on zolpidem withdrawal, and with zolpidem prescribed only for other indications without catatonia.

We aimed to include all randomised controlled trials (RCTs), case control studies, prospective and retrospective cohort studies, cross-sectional studies, case series and case reports where zolpidem was used to diagnose or treat at least one patient with catatonia. Additional searches were made to find full-text articles by searching online on Google Scholar, University and NHS OpenAthens accounts, through ResearchGate, and emailing authors directly, if other strategies to obtain full-text articles were not successful. We also requested additional information from authors where incomplete information was available from publication(s) selected.

For each publication, data was independently extracted by N.A. and H.H., and S.W. was available to arbitrate, if there was any disagreement. The extracted data included the following: the citation; study design; patient number; age; sex; country of origin; publication year; diagnosis of underlying and co-morbid psychiatric/medical disorders; rating scales for catatonia; pre- and post-zolpidem catatonia rating scores; zolpidem dose (mg/d); and serum zolpidem levels (if available).

Improvement in catatonia with zolpidem was assessed on clinical description and/or rating scale scores and recorded under three broad categories.

1. remission (complete resolution of catatonia clearly described in the publication)
2. partial response (improvement in catatonia, but lack of resolution of symptoms), and
3. non-response (absence of an improvement in catatonia).

If a publication described improvement but without clear details of a complete absence of catatonia with treatment, then a partial response was noted.

The results from data extraction are tabulated in Tables 1 and 2 (and Table 3 in supplementary files). As all studies were either case reports or case series, meta-analysis and calculation of effect sizes were not attempted. Instead, issues with reporting biases as well as degree of certainty of the data are mentioned in the discussion section below.

**Table 1.** Summary of case reports (The abbreviations are expanded below the table; see further details of cases in Table 3 in supplementary file 1).

Year	Authors and country	Cases	Age and sex	Psychiatric and medical diagnoses (in addition to catatonia)	QA score	Treatment details (including any BZ)	Catatonia measure(s) (score)	ZM as a challenge test^a^	ZM for treatment	Alone or with BZ^b^	Dose^c^	Response to ZM^d^
1995	Mastain et al.^23^ France Added details^24^	1	56 F	None except catatonia	7	Resistant to ECT, LZM, AZM, and zopiclone ZM 10mg tried	Scale based on BFCRS; Score (19/19)	Yes	Yes	Alone	10	Improved with “double blind” ZM; similar effect next day; reaching remission
1997	Rascle^25^ France	7	44 (mean) 4M, 3F	Mood disorder (4), SZ (1) GMC (2)	6	ZM threshold level 80-130ng/ml; response lasted for 3h	DSM	Yes	No	Alone	10	Positive response in 10-30min in 5/7 with remission; non-response in 2/7
1997	Thomas et al.^26^ France	1	21 F	S-AD (1^st^ E)	7	ZM 1m after HPL stopped; ZM level 90 ng/ml and relapsed at <90 ng/ml	DSM BFCRS	Yes	No	Alone	10	Improved in 15min; remission for 4h; similar response 24h later
1997 1999	Zaw et al.^27,28^ UK	1	14 M	ASD, psychotic depression	6	IV amytal – no response; ZM test; ZM level 0·17mg/L, falling to half at 2h	Roger’s scale DSM	Yes	No	Alone	7.5	Score from 27 to 18 at 2h, and to 10 at 4h, with partial response; Improvement for 24h
2006	Seetharam and Akerman^29^ US	1	49 M	Post-liver transplant catatonia Query delirium Febrile	3	ZM + OZM (small dose); resolved in 2w with OZM	None	Yes	No	With BZ	Unclear	Partial response within 24h with OZM and ZM
2006	Kocmur and Vodopivec^30^ Slovenia	1	54 F	Psychotic depression; past “extrapyramidal syndrome”	4	CBZ 600mg/d + ZM	None	No	Yes	Alone	10-20	Partial response 1w later
2007	Cottencin et al.^16^ (Case 1 of 12) France	1	22 F	Past school phobia; “melancholic delusions”; operated for a benign posterior fossa tumour; 1-week post-op onset of catatonia	5	ZM test 10mg LZM 7.5mg/d later; LZM stopped on day 20 with no recurrence	None	Yes	No	Alone	10	Partial response in 30min
2007	Cottencin et al.^16^ (Case 2 of 12) France	1	86 F	HT, past stroke, CRF, post-op dialysis; possible delirium	5	AZM 1.5mg/d; ZM test; Later LZM 1.5mg/d; resolution with LZM in 24h; later continued	None	Yes	No	With BZ	10	Partial response in 30min. Query also on AZM
2007	Cottencin et al.^16^ (Case 6 of 12) France	1	70 F	MDD PD	5	ZM test; later LZM 7.5.mg/d for 10d with remission	None	Yes	No	Alone	Unclear Query 10	Partial response in 30min
2007	Cottencin et al.^16^ (Case 7 of 12) France	1	22 F	ED, MDD, Pulmonary TB, planned lung resection	5	ZM test; later IV CZM; Response to oral LZM in 3-6w	None	Yes	No	Alone	Unclear Query 10	No response except vomiting
2007	Cottencin et al.^16^ (Case 10 of 12) France	1	68 F	Psychotic depression PAHT Disorientation in time and space	5	HPL stopped; ZM test; later LZM, with remission after 8d	None	Yes	No	Alone	Unclear Query 10	Partial response in 30min
2007	Cottencin et al.^16^ (Case 11 of 12) France	1	59F	Depression Confusion Fahr’s disease	5	ZM test; later LZM– stopped over several months	None	Yes	No	Alone	Unclear Query 10	Partial response in 30min
2007	Cottencin et al.^16^ (Case 12 of 12) France	1	50 F	Liver CA with embolization Alcoholic cirrhosis Possible delirium	5	ZM test tried; LZM continued later with improvement	None	Yes	No	Alone	Unclear Query 10	Non-response except sleep in 30min; next day improvement
2007	Thomas et al.^24^ France	20	NA	Mood disorder (12), GMC (3), SZ (3), idiopathic (2)	6	ZM test: all responded; correlation with ZM level of 80 to 150 ng/ml; later 15 responded to LZM	BFCRS (French translation)	Yes	No	Alone	10	Mean time to response: 32min Partial response lasted 185min (mean)
2008	Coconcea^31^ (Case 1) US	1	65 M	S-AD	2	Resistant to ECT and BZ; ZM tried next	None	No	Yes	With BZ	30 (10 TDS)	Dramatic partial response
2008	Coconcea^31^ (Case 2) US	1	23 F	SZ	2	Partial response to BZ ZM tried next	None	No	Yes	With BZ	Unclear	Marked partial response, relapse on non-concordance
2009	Cottencin et al.^17^ France	1	54 F	BaD; alcohol and GABAergic substance withdrawal prior to onset of catatonia HT, disorientation in time/space	4	ZM test Good response in 3d to LZM after ZM	BFCRS (40)	Yes	No	Alone	10	Partial response in 40min (score from 40 to 6); symptoms back at 3h (score 20)
2012	Bation et al.^32^ France	1	35 M	Psychotic depression with mood incongruent delusions, SLE, cerebral palsy	4	ZM 7.5mg 6 times a day Changed to AZM - sedation above 3mg/d	NCRS	No	Yes	Alone	45	Partial improvement of mutism/stupor; stopped due to AEs
2013	Isomura et al.^33^ Japan	1	69 F	Frontotemporal dementia	5	No response to zopiclone, AMD and BZ Effect sustained for 2y with ZM (remission)	BFCRS (26)	Yes	Yes	Alone	5	Response in 30min; lasted for few hours (score from 26 to 3)
2013	Peglow et al.^34^ US	1	46 M	S-AD, query NMS	6	No effect with 6 ECTs; dantrolene, AMD, bromocriptine Agitation with LZM; ZM tried alone; discharged on ZM + divalproex	None	Yes	Yes	Alone	30-40	Partial response on 5th day with ZM 10mg QDS, remission in 4w
2014	Appu & Noetzel^35^ US	1	16 F	Psychosis, ANMDARE, left ovarian teratoma, MCS	5	HPL, immunotherapy, plasmapheresis, tumour resection, steroids ZM on day 42; almost recovered in 2m, complete recovery in 6m	None	Yes	Yes	Alone	10	Improved, weaned off 3w after discharge Partial response
2014	Hlal et al.^36^ Morocco	1	24 F	SZ, NMS-like picture	6	Worsening within 2d after OLZ; no difference with DZM for 4w; then ZM + DZM	None	Yes	Yes	With BZ	12.5-25	12.5mg – good rapid response; didn’t last for >2-4h; later 12.5mg BD; remission in 1m
2015	Rajagopal^37^ India	1	71 F	Depressive episode Onset 1w after amiodarone	4	Amiodarone stopped ZM + CZM; stopped 1w later when in remission	None	Yes	Yes	With BZ	5	Partial response 1d after ZM + CZM; later remission
2015	Javelot et al.^38^ France	1	32 M	SZ, 3 episodes of catatonia	5	1^st^ E: ZM + LZM + OLZ 2^nd^ E: ARP, later RIS and LZM; partial response to ZM; stopped after 2w; improved on LZM 3^rd^ E (2y later despite LZM): amisulpride, later OLZ ZM + LZM – good response; gradually discontinued over 10d – no relapse 1y later	None	Yes	Yes	With BZ	30	Partial response to ZM+ LZM 1^st^ E: ZM stopped after 48h Relapse of catatonia 2m after stopping ZM 3^rd^ E: good response to ZM 30mg/d
2015	Bram et al.^39^ France	1	60 F	Pernicious anaemia, HT, sleep apnoea, hypothyroidism	4	ZM test; later LZM for 60d but relapse, adding B12 led to remission with no relapse at 1y	MINI/other scales, none for catatonia	Yes	No	Alone	10	Remission in 30min
2015	Ellul et al.^40^ France	1	27 M	ASD, MDD, query SZ	4	ZM test; then LZM with no improvement; then AMD led to marked improvement	BFCRS (46)	Yes	No	Alone	10	No response
2015	Jaimes-Albornoz et al.^41^ (Case 1 of 2) Spain	1	65 F	Fronto-temporal dementia; gastrostomy for food refusal	4	LZM 2.5mg + ZM 10mg Perseveration and mannerisms remained	BFCRS (32)	No	Yes	With BZ	10	Partially improved in 1w with LZM + ZM
2016	Rodgers and Arciniegas^42^ US (Case 1 of 3) (Case 2 of 3) (Case 3 of 3) Additional information from authors	1	Teenage F	Severe traumatic brain injury with MCS	4	AMD + Gabapentin ZM test	BFCRS (10) CRS-R (12)	Yes	Yes	Alone	5-10	Partial response Continued treatment helped improvement
		1	Middle age M	Multiple haemorrhagic strokes MCS	4	AMD 100mg BD; ZM Discharged to a nursing facility for ongoing care	BFCRS (5) CRS-R (16)	Yes	No	Alone	5-10	Non-response at 5mg, sedation at 10mg
		1	Middle age M	Hypoxic brain injury MCS	4	AMD 100mg BD; ZM test; then treatment transitioned to LZM Discharged home with intensive caregiving	BFCRS (23) CRS-R (4)	Yes	No	Alone	5-10	Partial response both doses
2016	Amorim and McDade^43^ US	1	73 F	Ovarian teratoma, DM type II, AF, HT, hypertensive encephalopathy, Inclusion body myositis, fluctuating confusion	6	12 ECTs; partial response to later ECTs; no response to tumour resection, BZ, APs, immunotherapy. ZM for sleep	None	Yes	Yes	Alone	5	Quick response to ZM; lasted for 6-10h Later less (partial) response, with full effect lasting no more than 4-6h
2016	Bastiampillai et al.^44^ Australia	1	59 F	BaD	6	LZM led to AEs, with no improvement though score from 18 to 12; no response to 24 ECTs; ZM followed by extended ECTs: led to complete resolution	BFCRS (18)	Yes	Yes	Alone	5	Partial response in 15min; resolution (score from 18 to 4) in 1h but relapse in 5-6h. Similar transient partial response to rechallenge
2019	Kapulsky et al.^45^ US	1	22 M	SZ, clozapine withdrawal catatonia, past NMS	4	Little benefit with ECTs; ZM on day 49; restart of clozapine helpful	None	No	Yes	Alone	5	ZM for 2d - AEs; ZM stopped; non-response
2019	Yamaguchi et al.^46^ Japan	1	74 M	Severe depression and anxiety; past catatonia, subclinical Cushing’s, HT, pituitary micro-adenoma	4	OLZ stopped; ZM tried; surgical removal of pituitary adenoma; no recurrence of catatonia	BFCRS (21) MMSE (21)	No	Yes	Alone	10	Marked response (score from 21 to 2 in “a few days”); in remission
2019	Sayadnasiri and Rezvani^47^ Iran	1	65 F	Frontotemporal dementia, MRI: left frontal atrophy, HT	6	Score from 18 to 9 with LZM but still mute despite adding MEM ZM 5mg added to LZM + MEM; later ZM 10mg BD LZM and ZM stopped due to AEs but no recurrence after 2m	BFCRS (18) MMSE 18 Letter frequency test	Yes	Yes	With BZ	5-20	Dramatic improvement in speech 20min after ZM added to LZM + MEM; effect lasted for 2-3h ZM 20mg + LZM + MEM: partial response
2019	Baptista and Choucha^48^ France	1	25 F	Psychosis, SLE with ANA Abs, AP-related catatonia	6	AP-related catatonia; HPL stopped + LZM: resolution of catatonia LZM stopped as worse agitation; remission with ZM; symptoms back with loxapine, but ZM rechallenge tried. Also, valproate for mania. DZM not helpful; ZM 10mg continued for >3y to prevent relapse	BFCRS (20)	Yes	Yes	Alone	10	Resolution in 30min; lasted 3h Rechallenge with complete resolution
2019	Ryan-Michailidis et al.^49^ US	1	22 M	Cardiac arrest with brain hypoxia, status asthmaticus	5	ZM response lasting more than 3-4h No other details of treatment	BFCRS (28) GCS (10) DSM-5 catatonia	Yes	Yes	Alone	5-10	Partial response to 5mg in 1h, with further improvement to 10mg (score from 28 to 2)
2020	Kumar and Kumar^50^ (Case 1 of 2) India	1	15 F	Psychosis of 1m duration with catatonia	7	Positive but transient result with LZM over 2d ZM tried; discharged on ZM + OLZ; remained well 2m later	BFCRS (20)	Yes	Yes	Alone	10-30	Partial response in 30min after ZM 10mg Remission in 3d with ZM 30mg/d
2020	Kumar and Kumar^50^ (Case 2 of 2) India	1	14 M	Catatonia	6	Improved on LZM (score from 22 to 18) ZM tried, discharged on ZM 12.5mg TDS	BFCRS (22)	Yes	Yes	Alone	30-37.5	Partial response in 30min following ZM test – no AEs; remission 2m later - ZM tapered and stopped
2021	Almeida et al.^51^ (Case 2 of 4) US	1	45	BaD	3	LZM + ZM: no effect; brief response to CPZ; score 25 to 0 with bilateral ECT	BFCRS (25)	Yes	No	With BZ	Unclear	No benefit with ZM and LZM
2021	Bean et al.^52^ UK	1	73 M	ASD, past depression, later SZ diagnosis considered	2	Response to LZM; later stopped due to AEs 35 ECTs, ARP, ZM in week 37-39, with no recent BZ since week 19 15 more ECTs, DZM, ARP; died after 2y	BFCRS (16)	No	Yes	Alone	Unclear	No improvement with ZM over 2w or follow-up over 16m
2021	Mahgoub et al.^53^ US	1	26 M	BaD, past NMS, relapse of paranoia	4	LZM, DZM and ZM didn’t help; ECT, clozapine and valproic acid helpful	BFCRS (14)	No	Yes	Query, with BZ	10	No benefit with ZM – no details whether given separately or jointly with BZ
2023	Wendt et al.^54^ US	1	78 M	MDD, CAD, CABG, cerebral aneurysm, stroke, vascular dementia	7	No change with multiple trials of IV LZM ZM trial 23h after unsuccessful IV LZM ZM stopped after 1m	BFCRS (24)	No	Yes	Alone	30	Score 21 after 2 doses of ZM 10mg TDS, and to 16 after total 70mg; then ZM 5mg TDS, score 7 (partial response). Rechallenge 9m later (score 20); good response to ZM 5mg TDS. Later reduced to 7.5mg/d - score 3 (partial response)
2023	Cruzado and Hurtado-Llamo^55^ Peru	1	45 F	SZ, type II DM (decompensated), query NMS, aspiration pneumonia, HT	4	No response to BZ, ZM on day 51, with ARP 15mg and MEM 20mg Maintained improvement at 1m	BFCRS (30)	No	Yes	Alone	20-30	Responded to ZM 20mg + ARP + MEM in 2d (no BZ); score from 30 to 11 in 4d; and then 6 in 5d (partial response)
2023	Bouaziz et al.^15^ France	1	70 F	SZ, early NPH	4	ECTs led to remission but stopped; ZM + LZM tried; tDCS later	BFCRS (40)	No	Yes	Query, with BZ	30	No response to ZM + LZM; unclear whether together or not
2023	Greenstein et al.^56^ US	1	65 F	OCD, Tourette’s, anxiety, peritoneal adeno-CA, post-op delirium, acute cerebellar infarct, query NMS, query autoimmune encephalitis	4	ZM 10-30mg on day 39, with LZM, AMD; LZM reduced to 8mg/d due to delirium Finally on clomipramine 50mg BD, LZM 1mg TDS, ZM 10mg/d	None	No	Yes	With BZ	10-30	Partial response to ZM 30mg + LZM + AMD + plasmapheresis 5d after starting ZM Remission on day 70 (31d after ZM)
2023	Spiegel et al.^57^ US	1	21 F	Left ovarian teratoma ANMDARE with psychosis, MCS, orofacial dyskinesias, dysautonomia	6	No response to tumour excision, IV IGs, plasmapheresis; ZM on Day 25; on Day 50 discharged to a rehab facility on ZM	BFCRS (23) CRS-R (15)	Yes	Yes	Alone	5-20	Remission in 8d Score dropped from 23 to 2 at discharge
2023	Bonomo et al.^58^ US	1	Middle age F	PTSD, psychosis past cocaine misuse Hypothyroidism Rabbit syndrome	7	No response to oral CZM and LZM ZM tried; LZM stopped and ZM 10mg TDS continued for treatment	None	Yes	Yes	With BZ	30 (10 TDS)	Response after first dose; also, on LZM initially; remission in few days to ZM 10mg TDS without AEs
2024	Parasher et al.^59^ India	1	20 M	SZ, large arachnoid cyst in posterior fossa behind cerebellar hemispheres with no mass effect	4	Good response to LZM but no change with LZM + APs Score from 12 to 9 with LZM, from 9 to 8 on adding ZM 30mg, and to 0 on adding ECT; relapsed when ECTs stopped despite ZM + OLZ; weekly ECT helpful	BFCRS (12)	Yes	Yes	Alone	30-40	Minimal response with ZM (score dropped from 9 to 8); recorded as non-response
2024	Yakubu et al.^60^ UK	1	70 F	TRD (numerous ADs, lithium, anti-anxiety, and APs)	5	No response to 15 ECTs, limited response to LZM; both stopped; ZM 10mg tried; then 10mg QDS with good improvement - continued + venlafaxine with marked improvement	BFCRS (17)	Yes	Yes	Alone	10-40	Partial response in 30min to ZM 10mg (score 17 to 6)
2024	Palma and Rayyan^61^ US	1	55 F	Past S-AD or BaD, CKD, acute hypoxic respiratory failure, query vasculitis	5	Steroids, plasma exchange; ZM + “psychiatric medication”	BFCRS (14)	Yes	Yes	Alone	10-15	Partial response to ZM 10mg in 30min (score 14 to 7); later ZM 5mg TDS
2024	Veluri et al.^62^ US	1	48 F	MDD	5	Improved with LZM + bupropion in 4d; ZM + LZM tried; relapsed after stopping ZM after a few weeks and tapering LZM Managed with LZM + ZM + MEM + AD	BFCRS (14)	No	Yes	With BZ	10 (5 BD)	ZM + LZM reduced score from 14 to 6 (partial response)
2024	Midkiff et al.^63^ Case 1 of 2 US	1	77 F	OCD, GAD, MDD Delirium	5	LZM led to resolution in 30min; delirium on Day 2 - LZM stopped, catatonia worse (score 8); CZM worsened delirium and catatonia (score 21); ZM tried	BFCRS (21)	No	Yes	Query, with BZ	15	Remission (score 0) in 24h to ZM 5mg TDS; delirium also improved; lack of clarity whether BZ were stopped
2024	Wadhwa et al.^64^ US	1	12 F	Psychosis with catatonia, ANMDARE, dysautonomia, hyperthermia	5	LZM 1.75mg IV 4 hourly - no benefit; steroids, IGs, rituximab given; no sustained improvement with ECT; LZM stopped; ZM tried next; weaned over several months	BFCRS (17)	No	Yes	Alone	7.5-25	ZM from 7.5mg to 22.5mg after 1w, and 25mg for 1m; score 17 to almost 0, score 5 after 2m Partial response

**Key: Abs:** Antibodies; **AMD:** Amantadine; **ANMDAR**: Anti-NMDA receptor; **ANMDARE**: Anti-NMDA receptor encephalitis; **AD(s)**: Antidepressant(s); **AEs**: Adverse effects; **AF**: Atrial fibrillation; **ANA**: Antinuclear antibodies; **AP(s)**: Antipsychotic(s); **ARP**: Aripiprazole; **ASD**: Autism spectrum disorder; **AZM**: Alprazolam; **BaD**: Bipolar affective disorder; **BD**: twice a day; **BFCRS**: Bush-Francis catatonia rating scale; **BZ**: Benzodiazepine(s); **CA**: Carcinoma; **CABG**: Coronary artery bypass graft; **CAD**: Coronary artery disease; **CBZ**: Carbamazepine; **CK**: Creatine kinase; **CKD**: Chronic kidney disease; **CPZ**: Chlorpromazine; **CRF**: Chronic renal failure; **CSF**: Cerebrospinal fluid; **CRS-R**: Coma Recovery Scale-Revised; **CZM**: Clonazepam; **d**: day(s); **DZM**: Diazepam; **DM**: Diabetes mellitus; **E**: episode; **ED**: Eating disorder; **ECT**: Electroconvulsive therapy; **EEG**: Electroencephalography; **EPSE**: Extrapyramidal side-effects; **F**: female; **GAD**: Generalised anxiety disorder; **GMC**: General medical conditions; **h**: hours; **H/o**: History of; **HPL**: Haloperidol; **HT**: Hypertension; **IGs**: Immunoglobulins; **IM**: Intramuscular; **IV**: Intravenous; **LZM**: Lorazepam; **OCD**: Obsessive compulsive disorder; **M**: Male; **MCS**: Minimally conscious state; **MDD**: Major depressive disorder; **MEM**: Memantine; **MI**: Myocardial infarction; **m**: month(s); **min**: minute(s); **MMSE**: Mini-mental state examination; **MRI**: Magnetic resonance imaging; **NA**: Not available; **NCRS**: Northoff catatonia rating scale; **NG**: Nasogastric tube; **NMS**: Neuroleptic malignant syndrome; **NPH**: Normal pressure hydrocephalus; **OLZ**: Olanzapine; **OZM**: Oxazepam; **PAHT**: Pulmonary artery hypertension; **PD**: Parkinson’s disease; **PO**: per oral; **PTSD**: Post-traumatic stress disorder; **QDS**: four times a day; **RIS**: Risperidone; **S-AD**: Schizoaffective disorder; **SLE**: Systemic lupus erythematosus; **SZ**: Schizophrenia/schizophreniform disorder; **tDCS**: transcranial Direct Current Stimulation; **TB**: Tuberculosis; **TDS**: three times a day; **TRD**: Treatment resistant depression; **TSH**: Thyroid stimulating hormone; **w**: week(s); **y**: year(s); **ZM**: Zolpidem.

^a^Challenge test includes cases where there is a response noted to the first single dose of zolpidem.

^b^Includes cases where zolpidem was used in a setting where benzodiazepines were also prescribed at the same time and timeline separating zolpidem and benzodiazepines wasn’t clear.

^c^Oral zolpidem dose (mg).

^d^If a case described improvement but without clear details of a complete absence of catatonia with treatment, then a partial response was noted.

**Table 2.** Frequency and percentage of response.

​	Frequency (n) and percentage (%)	Number of cases (n)	Positive response* n (%)	Remission n (%)	Partial response n (%)	No response n (%)
All cases (either for challenge or treatment)		79	67 (84.8)	19 (24.1)	48 (60.8)	12 (15.2)
​	Without co-prescribed benzodiazepines	63	54 (85.7)	15 (23.8)	39 (61.9)	9 (14.3)
​	With co-prescribed benzodiazepines	16	13 (81.3)	4 (25)	9 (56.3)	3 (18.8)
​	High and moderate quality cases	76	65 (85.5)	19 (25)	46 (60.5)	11 (14.5)
​	Medical/neurological disorders	30	24 (80)	7 (23.3)	17 (56.7)	6 (20)
As a challenge test		63	54 (85.7)	9 (14.3)	45 (71.4)	9 (14.3)
​	Without co-prescribed benzodiazepines	55	47 (85.5)	8 (14.5)	39 (70.9)	8 (14.5)
​	With co-prescribed benzodiazepines	8	7 (87.5)	1 (12.5)	6 (75)	1 (12.5)
As treatment		36	31 (86.1)	12 (33.3)	19 (52.8)	5 (13.9)
​	Without co-prescribed benzodiazepines	23	20 (87)	8 (34.8)	12 (52.2)	3 (13)
​	With co-prescribed benzodiazepines	13	11 (84.6)	4 (30.8)	7 (53.8)	2 (15.4)

*Positive response includes patients with either remission or partial response.

We calculated,

• summary statistics including mean, median, mode, and standard deviation for age, sex, and time of response.
• number of patients with
  • remission,
  • partial response, and
  • non-response
• underlying diagnoses,
• use of catatonia rating scales (e.g. the Bush-Francis Catatonia Screening Instrument – BFCSI, and the Rating Scale - BFCRS), with summary statistics regarding their use and scores, when available,
• use of zolpidem as a challenge test, for treatment, or for both treatment and challenge,
  • alone or
  • with benzodiazepines (if both were mentioned without clarity of timeline, both zolpidem and benzodiazepines were selected).
• spread of diagnoses in poor responders, and subsequent treatments tried, with outcomes.

The included publications underwent a quality assessment (QA), based on the GRADE framework,^65^ for evaluating their methodological quality.^66^ These eight questions include one question on selection of patients, two questions on ascertainment of exposure and outcome, four questions on causality, and one question of level of detail in reporting of the case. For ascertaining sufficient detail for this systematic review, age, sex, diagnosis of catatonia, psychiatric diagnosis, zolpidem initiation, and change in catatonia were the desired minimum information, though cases with less information were also included to maximise data collection. These 8 items are scored either as 0 or 1 on domains of patient selection, and the total score (out of 8) was considered to determine quality of each paper (low: 0-2; moderate: 3-5; high: 6-8). For each publication, this process was completed independently by H.H. and N.A., and with arbitration by J.T., if there was disagreement.

## Results

We identified 79 cases describing zolpidem use in diagnosis and/or treatment of catatonia, across 44 publications between 1995 and May 2025. Information from these 6 case series and 38 case reports and their QA is summarised in Table 1.

A PRISMA flowchart is shown in Figure 1.

**Figure 1.**
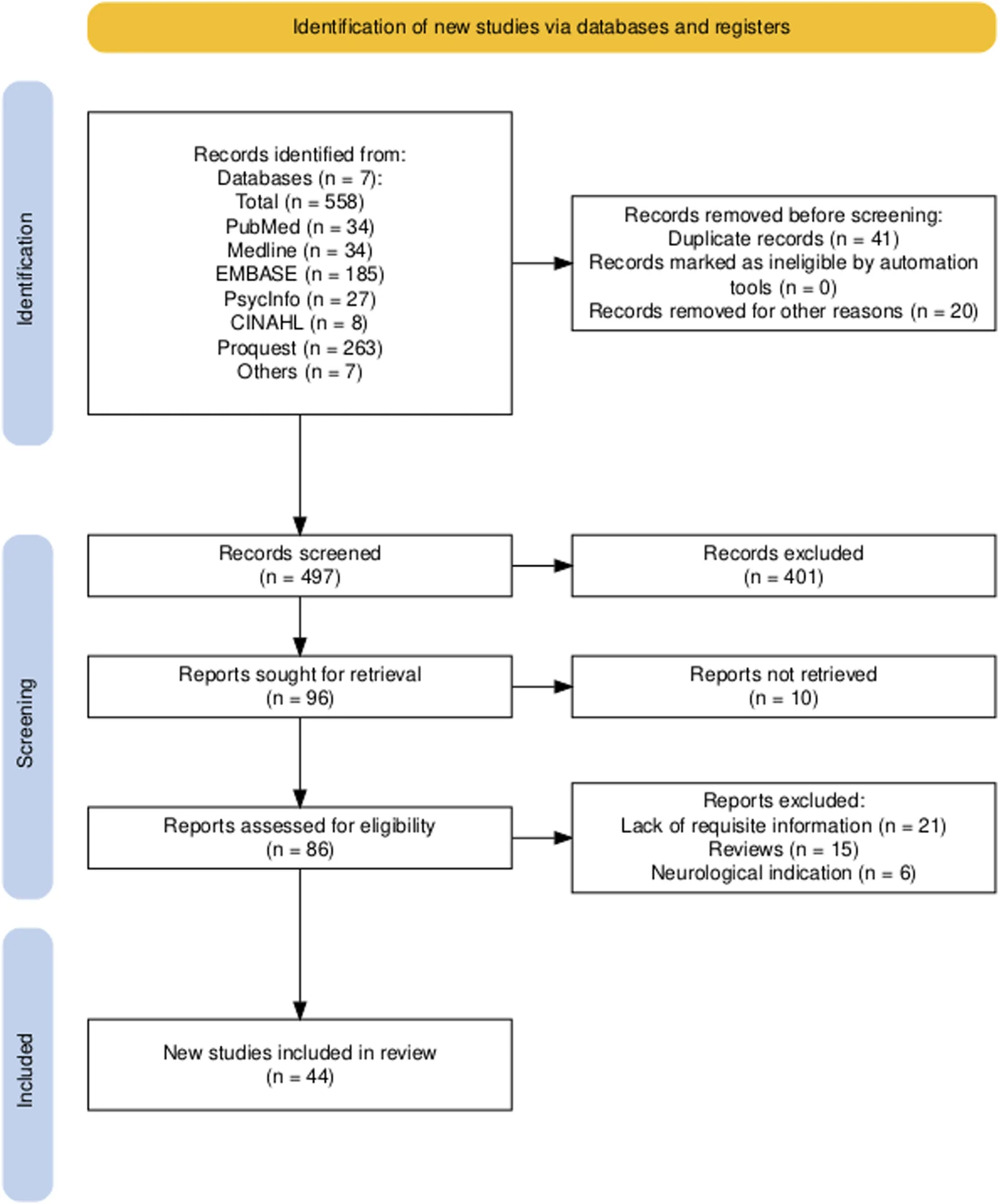
Flowchart of study selection (created using the PRISMA flow diagram tool)^67^

Of the 79 cases (Tables 1 and 3), 76 (96.2%) were rated as either high (49) or moderate (27) on the QA assessment,^66^ with 3 rated as low. The largest number of cases (43; 54.4%) originate from France, including the first report in 1995. The next largest number of reports is from the US (21).

Overall, 67 patients (84.8%) showed a positive response of catatonic symptoms to zolpidem (Table 2). Out of these 79, 19 (24.1%) achieved remission of catatonia, 48 (60.8%) had partial response, and 12 (15.2%) showed no response.

We used sensitivity analysis by excluding low-quality cases. When only considering high or moderate quality cases (76 cases), 65 (85.5%) showed a positive response, with 19 (25%) reaching remission, which is not dissimilar to analysis of all cases (Table 2).

There are 63 case reports of zolpidem used as a challenge test, with 36 cases using it for treatment of catatonia. When zolpidem was used as a challenge test, the number of responders was similar (85.7%), though with a lower percentage (14.3%) reaching remission with 71.4% partial responders and 14.3% non-responders. The number of those reaching remission was higher (33.3%) when zolpidem was used as treatment though total number of responders (86.1%) was similar to that for the challenge test.

Benzodiazepines were co-prescribed in 16 (20.3%), with little difference in number of responders (81.3%) as compared to when zolpidem was used alone (85.7%). (Table 2).

Information about age and sex was available in 60.8% and 74.7% of cases respectively. Zolpidem was used in a wide range of ages (12-86 years), with a mean of 46.5 years ± 22.3 SD (median 49.5 years). There were several reports in adolescents^27,28,35,42,50,64^ as well as in older adults above the age of 65.^15,16,33,37,43,52,54,60,63^ Nearly two-thirds of patients were female (64.4%). The response rate was higher in women (88.6% vs 64.7% in men), with 31.4% reaching remission (17.6% in men) and 57.1% being partial responders (47.1% in men).

Amongst 12 non-responders, information about diagnoses was available in only 10 patients (medical/neurological aetiology 60%, mood disorder 50%, schizophrenia 30%, and other categories 30%). These numbers do not add up to 100% as half the patients had co-morbid conditions. Some of the non-responders subsequently showed good response to ECT (4),^15,51,53,59^ ECT followed by clozapine and valproic acid (1),^53^ ECT followed by tDCS (1),^15^ lorazepam (1),^16^ clozapine (1),^45^ and amantadine (1).^40^ One patient died of sepsis^52^ and another patient was discharged to a nursing facility,^42^ with no improvement. One patient showed a delayed improvement the next day, following an initial negative response to zolpidem.^16^

A reduction in catatonia by at least 50% on the BFCRS, usually 30 minutes after administration of 10mg oral zolpidem, has been used in confirming diagnosis of catatonia as evidence of a positive response.^16,68^ The mean dose of zolpidem in this systematic review was 14.7mg (mode and median both 10mg). The lowest and highest effective doses were 5mg/d^33^ and 45mg/d^32^ respectively, without significant side-effects reported.

In 42 (53.2%) cases, where this data was available, the mean time to response was 30.2 minutes (range 15-60 minutes). There were two outliers where response occurred after 1 day and 1 week respectively, but it is difficult to attribute response to zolpidem in these cases as zolpidem has a short half-life.

Only 60.8% of reported cases utilised the BFCRS, with 30.4% not reporting use of any catatonia rating scale. In 27 cases, where BFCRS scores were reported, the mean BFCRS score was 22.15±9.39 SD (median 21, mode 14, range 5-46). 44.3% (35/79) of patients had an underlying mood disorder, with schizophrenia (15.2%), schizoaffective disorder (5.1%), and other categories (15.2%) making up the rest. 44.3% (35/79) had an associated medical/neurological condition.

## Discussion

This systematic review examines the evidence for the use of zolpidem in diagnosis and treatment of catatonia. In the absence of any published RCTs, we reviewed the published literature of 79 cases, consisting of single case reports and small case series described in 44 publications.

Our systematic review provides further corroboration for the efficacy of zolpidem in management of catatonia. The current review describes more than double the number of cases included in a previous review published in 2024,^69^ from 35 to 79. Our larger dataset includes a wide range of ages, varying from 12 years to 86 years, with reports in adolescents^27,28,35,42,50,64^ as well as in patients above the age of 65.^15,16,33,37,43,52,54,60,63^ The additional cases allow for a more detailed sensitivity analysis excluding low-quality reports (based on the GRADE framework^65^), and by separating zolpidem prescribed alone and alongside benzodiazepines. These cases are described in a summary form in Table 1, with a detailed account provided in Table 3 which is included as Appendix 2 in the supplementary materials.

Our review also extends the examination of the data by defining the outcomes for zolpidem use in catatonia as remission, partial response and non-response. The use of zolpidem in catatonia as a challenge test is separated from its use as treatment (or use as both) when reporting on efficacy in each case. We also report on the mean, mode and median dose of zolpidem used in the included cases, and found a trend towards a gender difference, favouring women, in zolpidem response in catatonia.

In this review, a positive response to zolpidem was noted in 84.8% of patients presenting with catatonia which is not dissimilar to the 80% response rate reported previously.^69^ These results remained robust across sensitivity analyses excluding publications with low-quality evidence and cases where zolpidem was co-prescribed with benzodiazepines. The number of responders were not very different whether zolpidem was used either alone (85.7%) or with benzodiazepines (81.3%), and this similarity held when analysed separately for use of zolpidem as a challenge test or for treatment. It is interesting to note that the overall response rate to zolpidem in catatonia was higher in women with 88.6% showing a positive response as compared to 64.7% of men; 31.4% of women reached remission with zolpidem as compared to 17.6% in men. Whether this is a real difference or related to some inherent bias from examining case reports and case series remains to be seen.

The overall total number of responders were also similar when zolpidem was used either as a challenge test (85.7%) or for treatment (86.1%). However, the number of partial responders dropped from 71.4% when used as a challenge test, to 52.8% when used for treatment, with a concomitant increase in number of patients reaching remission during treatment with zolpidem (33.3% as compared to 14.3% when used as a challenge test). The number of non-responders remained similar (13.9% for treatment and 14.3% for challenge test). This seems in line with the short half-life of zolpidem with a longer treatment duration helpful in getting better response in treating catatonic symptoms. Further improvement in catatonia was noted in some cases when zolpidem was continued for a longer period (e.g.,^23,33,34,36,37,49,50,54^) and/or dose was further increased (e.g.^34,36,37,49,50,54,55,57,60,64^) though this was not always the case (e.g.,^38,42,43,59^). A few reports^23–27^ describe usefulness of establishing therapeutic serum zolpidem levels of 80-150ng/ml, with one report of 170ng/ml,^27^ indirectly supporting the argument for extending the use of zolpidem in catatonia beyond a single-dose challenge test. Further studies of more than once daily use of zolpidem are needed to confirm where more doses are needed for an adequate response, in view of zolpidem’s short half-life.

### Medical/neurological diagnoses

It is encouraging that in those with medical/neurological disorders, often a reason for expecting non-response to benzodiazepines, 80% (24/30) showing positive response to zolpidem, with 23.3% reaching remission and an additional 56.7% showing a partial response (see Table 2).

There were five patients with possible auto-immune encephalitis,^35,43,56,57,64^ with four of these with ANMDARE (Anti-NMDA receptor encephalitis),^35,43,57,64^ with three related to an ovarian teratoma.^35,43,57^ Zolpidem was effective in treating catatonia in patients with ANMDARE in all four cases^35,43,57,64^ with reduced catatonic symptoms in three cases even when removal of ovarian teratoma, resection of which is the mainstay of treatment in ANMDARE with ovarian teratoma, did not lead to resolution of catatonia.^35,43,57^

Zolpidem use has been reported in neurological disorders including MCS (minimally conscious states), vegetative states, coma and Parkinson’s disease.^70^ In some reports, catatonia is described but not diagnosed^49,71^ and in others it is formally diagnosed.^35,42,49,57^ Identification of catatonia in patients with MCS and other neurological disorders may help in identifying those who may respond to zolpidem (e.g.,^35,42,57^), lorazepam and/or ECT.

Physicians and psychiatrists are often reluctant to prescribe lorazepam and/or other benzodiazepines in catatonia presenting with delirium, for the fear of worsening delirium and respiratory depression.^38,56,63^ Zolpidem may be a safer option in these patients^63^ though there is need for more evidence before this view can be supported.

### Symptom profile for response

The current catatonia guidelines^1,4^ highly recommend use of rating scales, such as the BFCSI and BFCRS^72^ which have screening utility,^1,4^ and can be used to support diagnostic assessment. However, only 60.8% of patients in this review used the BFCRS. There was little information about the individual items of the BFCRS, let alone progress with treatment.

There are some reports of improvement in verbal output. These may suggest a specific pro-linguistic zolpidem effect or the effect may simply be secondary to a non-specific improvement in catatonia.^16,24,47,71,73^ A report^73^ describes marked improvement in chronic post-stroke aphasia, in the apparent absence of catatonia, after 20 minutes of zolpidem administration. This was associated with a 30-40% increase in regional cerebral blood flow in Broca’s area, half an hour after zolpidem ingestion. There is also a case report of a 28-year-old man with a three-year history of traumatic intracerebral haemorrhage and associated semi-coma where a dramatic improvement in speech was seen 15 minutes after zolpidem use.^71^ Another report^34^ describes partial improvement in rigidity and mutism with zolpidem but without comment on any differential response. If zolpidem has a specific effect on improving verbal output, this has an important bearing on its use in catatonia, as there is some suggestion that lorazepam may not be as good in improving speech in catatonia.^74^

There is lack of clarity whether zolpidem works as well in excited catatonia with some reports describing a lack of effectiveness of zolpidem (and lorazepam) in catatonic excitement.^51^ There are case reports^48,75^ suggesting zolpidem may augment the antipsychotic effect of treatment in difficult-to-treat schizophrenia, though insomnia on zolpidem withdrawal may have contributed to worsening of psychosis.

### Adverse effects of zolpidem

We did not explore adverse effects of zolpidem as most case reports don’t describe these in detail (except, e.g., sedation at 45mg/d^32^). As zolpidem is taken orally, it is simpler to use and requires fewer precautions than injectable lorazepam.^16^ It is important that safety of both high-dose and longer-term zolpidem use is studied further, particularly in children/adolescents, and in elderly and frail patients who are likely to be more susceptible to adverse effects.^76^

### Place of zolpidem in catatonia treatment pathway

Benzodiazepines remain the first-line treatment for catatonia^1^ though not all patients respond, and this seems particularly true of catatonia in schizophrenia and chronic catatonia.^77^ There are very few studies comparing different benzodiazepines in treating catatonia^14^ and lorazepam is the most common benzodiazepine in clinical use. It is therefore not surprising to see more than 80% of patients in this review prescribed benzodiazepines at some point. It is interesting to note that parenteral lorazepam is difficult to obtain in France^16,17,19^ and there has been a recent shortage of lorazepam in the US.^18,20^

When benzodiazepines don’t work, the next step is often ECT,^1^ which is not always available,^15,30,50,55,62^ may be contraindicated,^54,56^ or declined by the patient.^16,62^ Only 22.8% of cases in this review describe ECT use in the treatment of catatonia. There are case reports of accidental improvement in catatonia when zolpidem was prescribed for improving sleep.^35,43^ Zolpidem was effective in many cases even when there was no or partial response to ECT^23,43,44^ and/or benzodiazepines.^23,33,34,36,38,43,44,48,50,54,55,58,63,64^ Zolpidem may be particularly useful when benzodiazepines^16^ and/or ECTs^30,55,62^ are not available, don’t work,^23,33,34,36,38,43,50,54–56,58,63,64^ or are considered inappropriate.^50,54,56,58,62^

Zolpidem seems to work both alone and in combination with benzodiazepines such as lorazepam,^38,41,47,56^ clonazepam^37^ and oxazepam.^29^ Interestingly, a few patients who responded to zolpidem, didn’t respond to lorazepam later and needed ECT, while other patients who didn’t respond to lorazepam showed a response to zolpidem.^23,24,56^ Lorazepam and zolpidem are both effective in catatonia unlike zopiclone, eszopiclone, alpidem, and zaleplon (e.g.,^23,33^). However, there are no RCTs comparing these drugs against each other, benzodiazepines (e.g., lorazepam), or placebo.

Zolpidem has a rapid onset of action (mean 30.2 minutes) and a short elimination half-life of 2.4 hours with no active metabolites. In comparison, lorazepam has a half-life of about 12 hours.^78^ Any adverse effects that may appear with zolpidem are likely to be short-lasting, often limited to sedation.^16^ This makes it easier to use but also may limit its value in long-term management of catatonia though three times daily dosing is a recommended option.^38^ Zolpidem is available in oral and sublingual forms but there is no parenteral zolpidem formulation. In the cases reviewed here, the lowest and highest effective doses were 5mg/d^33^ and 45mg/d^32^ respectively, which appears in line with recommendations of using zolpidem in the dose range of 7.5-40mg/d.^68^ Some patients need higher doses (e.g.^34,36,37,49,50,54,55,57,60,64,69^) and/or longer use (e.g.,^23,33,34,36,37,49,50,54^), ranging from 1 month^55^ to 2 years.^33^ It is unclear when to stop zolpidem if it helps in improving catatonia. This is not different to the lack of clarity regarding when to stop lorazepam in catatonia^4^ which is often prescribed for longer periods in clinical practice. It is difficult to conclude duration of treatment from this review as information regarding the time frame of stopping zolpidem after a positive response and ongoing remission was not always available.

The American Psychiatric Association (APA) catatonia resource document^4^ briefly mentions zolpidem, while the British Association for Psychopharmacology (BAP) guidelines (p. 339)^1^ suggest a diagnostic challenge with either lorazepam (stronger strength of recommendation: B) or zolpidem (next step strength of recommendation: C) when faced with an uncertain diagnosis of catatonia. Both guidelines emphasise the importance of treating the underlying cause of catatonia, while a judicious use of lorazepam and/or zolpidem helps with diagnosis and acute management. A large collection of case reports strengthens the evidence while waiting for placebo and/or lorazepam controlled RCTs of zolpidem in catatonia to be conducted.

## Strengths and limitations

This systematic review looked at 79 cases of zolpidem use in catatonia, over the last three decades. There has been a previous systematic review^69^ of 35 cases and our systematic review expands the evidence base of zolpidem use in catatonia. The strength of this review is inclusion of 79 cases of zolpidem use, both for diagnosis and treatment of catatonia.

It has been difficult to conduct RCTs of zolpidem (or even lorazepam) use, in catatonia, and therefore a systematic review of case reports and case series provides the best available order evidence on which to base clinical practice.^66^

Case reports and case series carry a risk of reporting bias.^79^ By necessity, there is a significant heterogeneity of diagnoses, comorbid diagnoses, use of catatonia rating scales, clear description of response, details of individual catatonic signs and their response to zolpidem, short and long-term adverse effects, and co-prescribed medication in this population. We used a quality assurance measure^66^ and analysed high and moderate quality reports separately which didn’t change the overall results of this systematic review.

An unavoidable limitation of our systematic review is that several patients with catatonia were prescribed benzodiazepines, in line with the current catatonic treatment guidelines.^1,4^ Therefore, we separated patients where zolpidem was used alone from those where benzodiazepines were co-prescribed (20.3%). If both zolpidem and benzodiazepines were mentioned without clarity about their timeline, it was presumed that these were co-prescribed and their data analysed in the zolpidem and benzodiazepine subgroup. The sensitivity analysis after exclusion of patients co-prescribed benzodiazepines at the same time showed similar number of responders.

One of the strengths of this review is the examination of completeness of catatonia response with zolpidem by dividing the responses into remission, partial response, and non-response. However, as not all publications clearly describe the use of rating scales before and after zolpidem use, and don’t clearly mention absence of catatonic symptoms after prescribing zolpidem, this introduces heterogeneity and causes problems in interpretation of data. Therefore, we followed the pragmatic rule of counting any response as partial response unless the authors clearly described an absence of catatonic symptoms clinically or with use of rating scales in their publication. This understandably leads to a potential underestimate of those reaching remission from catatonia but nevertheless allows for recognition that some patients can reach remission in their catatonic symptomatology with the use of zolpidem.

A few negative results with zolpidem are also published^80,81^ but without any further details about the dose and/or other clinical information. There are case reports of appearance of catatonia on abrupt withdrawal from long-term zolpidem use or other GABAergic substances,^17^ indicating a possible causal link.^82^ These reports are not included here unless relevant details were reported by the authors.^17,52^ The dataset for benzodiazepines and ECT is larger than that for zolpidem.^1,4,83^

The current review has attempted to include all positive and negative reports, wherever sufficient information was available about the clinical presentation of catatonia and use of zolpidem. In a few cases where there was an incomplete dataset, attempts were made to contact authors directly, where possible, to obtain more clinical information and to use language translation tools to assess articles in non-English language.

## Conclusions

This systematic review found zolpidem a promising agent as a diagnostic test and for treatment of catatonia worthy of more investigation and research, before its use becomes common practice. The current BAP guidelines^1^ recommend the use of lorazepam or zolpidem as a *challenge test* (p. 339), when there is uncertainty regarding a diagnosis of catatonia, though recognising the recommendation regarding zolpidem is weaker due to less evidence. The BAP guidelines^1^ do not make any recommendations for use of zolpidem in the *treatment* of catatonia.

Our review provides more evidence that zolpidem may potentially be used as an alternative to lorazepam both as a challenge test and a potential next step treatment of catatonia. For longer term management, treatment of underlying cause, use of ECT and/or benzodiazepines, and/or low dose antipsychotics (in cases with psychosis) is also needed. Its shorter half-life, ease of use of oral formulation, and quick time to response, may allow it to be used even earlier in the treatment pathway. Several patients show response with 10mg of zolpidem though others may need higher doses of up to 45mg daily. The efficacy of zolpidem appears linked with its positive allosteric modulatory effect on the GABA-A receptors with greater affinity for the α1 subunit of GABA-A.

These results should be used with caution clinically, as there is an inherent inability to generalise results from case reports and case series. There is need for further research, particularly randomised controlled trials, comparing zolpidem against placebo and benzodiazepines, and its safety data, to provide a safe and higher-order evidence for treatment of catatonia.

## Supplemental material

Supplemental material for A systematic review of case reports and case series of zolpidem in the diagnosis and treatment of catatonia: PRISMA Checklist Supplemental material for A systematic review of case reports and case series of zolpidem in the diagnosis and treatment of catatonia by Niraj Ahuja, Harry Hackett, Joseph Thorne, Stuart Watson, Richard Hamish McAllister Williams in Therapeutic Advances in Psychopharmacology

Supplemental material for A systematic review of case reports and case series of zolpidem in the diagnosis and treatment of catatonia: Table 3 Supplemental material for A systematic review of case reports and case series of zolpidem in the diagnosis and treatment of catatonia by Niraj Ahuja, Harry Hackett, Joseph Thorne, Stuart Watson, Richard Hamish McAllister-Williams in Therapeutic Advances in Psychopharmacology

## Data Availability

All data included in this publication is reported in a tabular form in the manuscript (see Table 1; expanded dataset available as Table 3 in Supplementary materials).*
