# Supplementary material for: A systematic review of case reports and case series of zolpidem in the diagnosis and treatment of catatonia
Source: Ther Adv Psychopharmacol. 2026 Sep 25;16:20451253261487038. doi: 10.1177/20451253261487038 (PMC13768662; doi:10.1177/20451253261487038)
Supplement: Supplementary file 2 [file sj-pdf-2-tpp-10.1177_20451253261487038.pdf]

Article title: A Systematic Review of Zolpidem in the Diagnosis and Treatment of Catatonia

Article title
A Systematic Review of Zolpidem in the Diagnosis and Treatment of Catatonia

Author name(s)
Niraj Ahuja[1,2], Harry Hackett[1,2], Joseph Thorne[1,2], Stuart Watson[1,2,3], Richard Hamish McAllister-Williams[1,2,3]

Affiliation(s)
[1]Cumbria, Northumberland, Tyne and Wear NHS Foundation Trust, Newcastle upon Tyne, NE3 3XT, UK
[2]Translational and Clinical Research Institute, Newcastle University, Newcastle upon Tyne, UK
[3]NIHR Newcastle Biomedical Research Centre (BRC), Newcastle upon Tyne, UK

Corresponding author address
Dr Niraj Ahuja
Consultant Psychiatrist
Regional Affective Disorder Service (RADS)
St Nicholas House,
St Nicholas Hospital
Jubilee Road
Gosforth
Newcastle Upon Tyne
NE3 3XT
UK

Corresponding author email address
niraj.ahuja@ncl.ac.uk

Article title: A Systematic Review of Zolpidem in the Diagnosis and Treatment of Catatonia

Supplementary File 1

Appendix 2. **Table 3. Case Report Details**
(The abbreviations are expanded below the table)

Year	Authors and Country	Number of cases	Age and Sex	Psychiatric diagnoses	Medical diagnoses	QA score	Treatment details	Catatonia measures (score)	Oral ZM dose (mg)[c]	Response to ZM[d]
**A1. ZM as a challenge test[a] alone**										
1997	Rascle [1]<br>France	7	44 (mean age)<br>4M<br>3F	7 cases: affective disorder (4), SZ disorder (1), GMC (2)<br>Catatonia	GMC in 2 cases	6	ZM threshold level 80-130ng/ml<br><br>Correlation between plasma ZM levels and response	DSM	10	Positive response in 15 minutes (mean; with a range of 10-30 minutes) in 5/7 with remission ("complete resolution of catatonic signs") (except in 2/4 patients with affective disorder)<br><br>Lasted 3h (2-5h)
1997	Thomas et al [2]<br>France	1	21 F	S-AD (1st episode)<br><br>Catatonia with oppositional behaviour, mutism, staring, posturing, food/drink refusal	None	7	ZM tried 1m after stopping HPL which worsened catatonia<br><br>Plasma ZM level 90 ng/ml – relapsed when levels below 90 ng/ml<br><br>Subsequently on AZM 6mg/d with remission	DSM<br>BFCRS	10	Marked improvement in 15 minutes with ZM 10mg; remission ("all her motor and behavioural signs resolved") lasted for 4h.<br><br>Trial repeated 24h later with a similar response
1997<br>1999	Zaw et al [3, 4]<br>UK	1	14 M	ASD, depression and psychotic symptoms<br>Catatonic stupor, mutism, rigidity, psychological pillow, waxy flexibility, mitgehen, posturing, grimacing, excitement	None	6	IV amytal 0.2g – no difference<br><br>Plasma ZM level peaked at 0·17mg/L, falling to half this value at 2h.<br><br>Imipramine 75mg/d for 6w – no response<br><br>ZM test<br><br>Subsequent good response to ECT<br><br>Discharged on Sulpiride 800mg/d and Lithium	Roger's scale<br>DSM	7.5	Good response to ZM test (7.5mg)<br><br>The catatonia score fell from 27 to 18 at 2h, and to 10 at 4h, with partial response<br><br>Improvement sustained for 24h
2007	Cottencin et al [5]<br>(Case 1 of 12)<br>France	1	22 F	Past school phobia<br>Catatonia with oppositional	Operated for a benign tumour in the posterior fossa	5	ZM test 10mg	None	10	Partial response in 30 minutes<br><br>LZM 7.5mg/d later; LZM stopped on Day 20 with no recurrence (remission

				behaviour, reclusion with apathy, negativism, refusal of food, automatic obedience<br><br>"Melancholic delusions"	1-week post-op					for treatment; "no psychopathological disorders were then noted")
2007	Cottencin et al [5] (Case 6 of 12) France	1	70 F	Major depressive disorder<br>Catatonia with food refusal, complete mutism, extreme psychomotor retardation, fixed gaze, opposition, negativism, verbal stereotypies, oppositional hypertonia	Parkinson's syndrome	5	Fluoxetine 20mg 8m back, benzerazide-levodopa 187.5mg/d, HPL 12mg/d, AZM, alimemazine<br><br>All stopped before ZM test	None	Unclear Query 10	Partial response in 30 minutes, including improvement in verbal fluency<br><br>Later LZM 7.5.mg/d continued for 10 days with remission of catatonia ("only depressive symptomatology remained")
2007	Cottencin et al (Case 7 of 12) France	1	22 F	Eating disorder symptoms during recovery from TB, MDD<br>Catatonia, refusal to eat, apathy, negativism, clinophilia, mutism, laconic replies, oppositional hypertonia, cogwheel rigidity	Pulmonary TB, planned lung resection	5	Isoniazid, rifampicin, pyrazinamide and ethambutol<br><br>ZM test<br><br>Later IV CZM 3-6mg/d: no response<br><br>IV LZM not available in France<br><br>Response to oral LZM 10mg/d (via nasogastric tube) instead in 3-6w	None	Unclear Query 10	No response to ZM test (10mg): vomiting
2007	Cottencin et al (Case 10 of 12) France	1	68 F	Psychotic depression, catatonia with grasping, echopraxia, oppositional hypertonia, automatic obedience, agitation, for 8m following death of brother	PAHT<br>Disorientation in time and space	5	HPL 2.5mg/d on admission: stopped<br><br>ZM test<br><br>Later LZM 4mg/d to 10mg/d, with remission after 8 days	None	Unclear Query 10	Partial response to ZM in 30 minutes
2007	Cottencin et al [5] (Case 11 of 12) France	1	59 F	Depression; past depression at 20<br><br>Catatonia with abulia,	Confusion<br>Fahr's disease	5	Worse on cyamemazine<br><br>ZM test	None	Unclear Query 10	Partial response to ZM in 30 minutes with improvement in confusion and much improved speech

				apragmatism, psychomotor retardation, poor spontaneous speech, alternating whispering and mutism, negativism			Later LZM 7.5mg/d – stopped over several months<br><br>APs stopped			
2007	Cottencin et al [5] (Case 12 of 12) France	1	50 F	Post-op catatonia with agitation, aggressiveness, verbal stereotypies, negativism, semi-mutism, echolalia, grasping<br>Alcohol dependence	Liver carcinoma with embolization, alcoholic cirrhosis<br>Possible delirium	5	ZM test tried<br><br>LZM 3mg/d continued later with improvement but with fluctuations	None	Unclear Query 10	Probably negative response except sleep in 30 minutes; next day improvement
2007	Thomas et al [6] France<br><br>The authors also cite Mastain et al 1998 with 10 cases but we could not locate the original article; therefore, these cases have not been included in this review.	20	NA	20 patients with catatonia<br>Mood disorder (12), GMC (3), acute SZ (3), idiopathic (2) – catatonia duration ranged from 2 days to 3y	GMC (3)	6	Correlation with a plasma ZM threshold of 80 and 150 ng/ml<br><br>ZM test: all responded<br><br>This was followed by LZM 4-12.5mg/d to which 15 responded; 5 patients with mood disorder needed ECT	BFCRS (French translation)	10	Mean time to response to ZM: 32 minutes<br><br>Response (defined as at least 50% reduction in catatonic symptoms on BFCRS) lasted for a mean of 185 minutes; taken as partial response in absence of fuller information
2009	Cottencin et al [7] France	1	54 F	Bipolar II disorder, alcohol withdrawal 3w earlier<br><br>Catatonia (after 48h of alcohol withdrawal) with fluctuating mutism, oppositional, akinesia, rigid/fixed posture, stereotypy, wandering	HT, abrupt stoppage of GABAergic substances (clorazepate and acamprosate), and cyamemazine, citalopram, nicardipine<br><br>Disorientation in time and space	4	ZM test<br><br>Good response in 3 days to LZM 15mg/d post-ZM<br><br>Discharged on valpromide 1200mg/d, nicardipine, LZM 5mg/d	BFCRS	10	Partial response to ZM in 40 minutes (BFCRS dropped from 40 to 6); symptoms back at 3h (BFCRS 20)
2015	Bram et al [8] France	1	60 F	Catatonia with psychomotor slowing, apathy, minimal changes of facial expression, poor spontaneous	Pernicious anaemia with mild vitamin B12 deficiency, HT, early menopause, sleep apnoea, hypothyroidism	4	ZM test<br><br>Later successfully treated with LZM 4mg/d for 60 days but relapsed and so B12 added which led to remission<br><br>No relapse at 1y follow-up	MINI and other scales but none for catatonia	10	Remission ("recovered from all her symptoms") in 30 minutes after ZM

				movements, waxy flexibility, motor stereotypy						
2015	Ellul et al [9]<br>France	1	27 M	High functioning ASD, MDD following bereavement, suspected SZ<br><br>Catatonia with verbigeration, motor stereotypies, mannerisms, ambitendency, posturing, catalepsy, waxy flexibility, negativism	None	4	Fluoxetine 20mg/d for 6w - no improvement, ARP 15mg/d for 4w - no better<br><br>ZM test<br><br>Then LZM 7.5mg for 3 days - no improvement<br><br>AMD 200mg/d - marked improvement (BFCRS 46 to 4 in 2w)	BFCRS (46)	10	No response to ZM
2016	Rodgers and Arciniegas [10]<br>(Case 2 of 3)<br>US<br><br>Additional information obtained from the authors	1	Middle age<br>M	Catatonia with immobility/stupor, staring, and stereotypy	Multiple strokes with haemorrhage in left middle cerebral artery territory, right thalamus, and anterior limb of right internal capsule, and MCS	4	AMD 100mg BD<br><br>ZM test<br><br>Discharged to a nursing facility for ongoing care	BFCRS (5)<br>CRS-R (16)	5-10	Equivocal response at 5mg and sedation at 10mg<br>CRS-R score unchanged at 16
2016	Rodgers and Arciniegas [10]<br>(Case 3 of 3)<br>US<br><br>Additional information obtained from the authors	1	Middle age<br>M	Catatonia with immobility/stupor, mutism, staring, posturing, grasp, grimacing, rigidity, negativism, gegenhalten, dysautonomia	Hypoxic brain injury and MCS	4	AMD 100mg BD<br><br>ZM test; then treatment transitioned to LZM<br><br>Discharged home with intensive caregiving	BFCRS (23)<br>CRS-R (4)	5-10	ZM produced temporary improvement in MCS (CRS-R score from 9 to 4, and then to 12) and reduced catatonia at both doses
A1: Total cases = 40										
**A2. ZM as a challenge test with benzodiazepines**[b]										
2006	Seetharam and Akerman [11]<br>US	1	49 M	"Complete" catatonia with echolalia, mutism	3 days post-liver transplant (for alcohol induced cirrhosis)<br>Suspected delirium<br><br>Febrile, no alcohol for 16m<br><br>Normal MRI<br>EEG suggestive of encephalopathy	3	OLZ (small dose for suspected delirium) and cyclosporine A stopped, mycophenolate and methylprednisolone continued<br><br>ZM (dose unclear) + OZM (small dose)	None	Unclear	Partial response within 24h with OZM and ZM<br><br>Resolved over 2w with OZM

2007	Cottencin et al [5]<br>(Case 2 of 12)<br>France	1	86 F	Catatonia with grasping, automatic obedience, gesture stereotypies, disinhibition	HT, past cerebellar stroke, CRF with atheromatous stenosis of the right renal artery, requiring angioplasty and insertion of a stent into the right renal artery<br><br>Post-op dialysis<br><br>Possible delirium with disorientation in time and space	5	AZM 1.5mg/d for anxiety and sleep<br><br>ZM test<br><br>Later LZM 1.5mg/d; resolution with LZM in 24h; then continued as treatment<br><br>Later depressive syndrome – responded to antidepressant and LZM in a few weeks	None<br><br>Sketchy details of catatonic syndrome	10	Partial response in 30 minutes to ZM (but it seems she was also on AZM at that time)
2021	Almeida et al [12]<br>(Case 2 of 4)<br>US	1	45	BaD<br>Excited catatonia with agitation, perseveration, motor stereotypies, posturing, gegenhalten, combativeness		3	IM HPL + LZM<br><br>Started on quetiapine (dose unclear) – catatonic symptoms<br><br>LZM 2mg IM QDS + ZM (dose not mentioned): no effect<br><br>Chlorpromazine 50mg IM-brief response<br><br>Improved with bilateral ECT (BFCRS 25 to 0)	BFCRS (25)	Unclear	No benefit with ZM and LZM
A2: Total cases = 3										
Total cases (A1 + A2) = 43										
**B. ZM as treatment alone**										
2006	Kocmur and Vodopivec [13]<br>Slovenia	1	54 F	Psychotic depression<br><br>Catatonia with rigidity, tremors, monotonous speech, motor stereotypies	Previous diagnosis of "extrapyramidal syndrome"; neurological symptoms on L-dopa	4	Stopped sertraline and OLZ 3w earlier<br><br>OLZ, Moclobemide and Sertraline didn't help; ECT not available<br><br>Carbamazepine 600mg/d + ZM	None	10-20	Partial response to ZM 1w later
2012	Bation et al [14]<br>France	1	35 M	Psychotic depression with mood incongruent delusions<br>Catatonia with mutism, stupor with	SLE, cerebral palsy with spastic tetraparesis	4	Prednisone, mycophenolate mofetil, fluindione for SLE, clomipramine 100mg, venlafaxine 150mg + OLZ 10mg which was changed to	NCRS	45	Partial improvement of mutism and stupor with ZM 45mg but stopped due to excessive daytime sedation<br><br>Scores not mentioned for ZM response

				alternating excitement, compulsive laughing			escitalopram 20mg + ARP 15mg<br><br>ZM 7.5mg 6 times a day<br><br>Then changed to AZM - sedation above 3mg/d<br><br>ECT started, developed fever after each of the 4 sessions but catatonic symptoms down by half (NCRS score dropped from 16/80 to 8/80 after 3rd ECT, and 5/80 after 2m)<br><br>ARP 15mg - resolution; focus on malignant hyperthermia			
2019	Kapulsky et al [15]<br>US	1	22 M	Paranoid SZ on clozapine for ~2y<br><br>Catatonia with rigidity, mutism, withdrawal, staring, echo-phenomena, posturing, negativism	Clozapine withdrawal catatonia with raised CK, past NMS with RIS	4	Initially BZ (query LZM) 28mg/d + bromocriptine 5mg/d (no response), benztropine IV on day 15<br><br>8 ECTs on day 30 (minimal benefit)<br><br>ZM tried on day 49<br><br>Restart of clozapine 175mg/d helpful	None	5	ZM 5mg for 2 days - more confused and dysregulated – stopped
2019	Yamaguchi et al [16]<br>Japan	1	74 M	Severe depression and anxiety; past h/o catatonia (ECT responsive), catatonia query after stopping OLZ 5mg/d	Subclinical Cushing's disease with a pituitary micro-adenoma, HT<br>MMSE 21/30	4	OLZ 5mg/d stopped<br><br>ZM test<br><br>Surgical removal of pituitary adenoma; no recurrence of catatonia since	BFCRS (21)<br>MMSE (21)	10	Marked response to ZM in a few days (BFCRS score dropped from 21 to 2 in "a few days"; MMSE improved from 21 to 24); in remission from catatonia ("catatonic symptoms were gradually relieved in a few days")
2021	Bean et al [17]<br>UK	1	73 M	ASD, past depression, later SZ diagnosis considered<br><br>Catatonia with mutism, agitation, stereotypy, incontinence	New onset HT<br>Negative antibody screen<br>Possible delirium	2	Mirtazapine, then RIS added to which sertraline was later added<br><br>Dramatic positive response to 2mg LZM test for doing a CT scan<br><br>LZM up to 0.5mg QDS for several days; later reduced and stopped due to severe sedation and falls<br><br>Bilateral ECTs 35, ARP	BFCRS (16)	Unclear	No improvement with ZM over 2w or follow-up over long admission (16m).

							ZM was tried in Week 37-39, with no recent BZ since week 19<br><br>15 more ECTs, DZM and ARP later<br><br>Patient died of sepsis after 2y.			
2023	Wendt et al [18]<br>US	1	78 M	MDD<br>Catatonia with mutism, staring, withdrawal, immobility, posturing, negativism, rigidity, declining oral intake<br><br>Past good response to ECT for TRD	CAD, CABG, cerebral aneurysm, stroke, vascular dementia	7	Duloxetine 20-40mg/d, Metoprolol, Tamsulosin, Aspirin, Atorvastatin<br><br>LZM IV multiple trials – no change<br><br>Couldn't give ECT due to physical comorbidities<br><br>ZM trial 23h after unsuccessful IV LZM trial	BFCRS (24)	30	BFCRS dropped to 21 after 2 doses of ZM 10mg TDS, and to 16 after 70mg total dose<br><br>Later dose reduced to 5mg TDS – BFCRS score dropped further to 7 (partial response). ZM stopped after 1m<br><br>Rechallenge 9m later when admitted again with BFCRS score 20<br><br>Good response to ZM 5mg TDS. later reduced to 7.5mg/d - BFCRS score of 3 (partial response)
2023	Cruzado and Hurtado-Llamo [19]<br>Peru	1	45 F	Paranoid SZ<br><br>Catatonic stupor with agitation, reduced food intake, fluctuating mutism, negativism, grimacing, stereotypies	HT, type II DM, query NMS with high dose APs (raised CK), aspiration pneumonia, decompensated DM	4	Depot APs for 12y, HPL decanoate at admission, HPL, CZM, RIS, CPZ, DZM, OLZ, ziprasidone<br><br>No response to BZ<br><br>ECT couldn't be given due to lack of bedspace in the inpatient unit<br><br>ZM tried on day 51, jointly with ARP and MEM<br><br>Maintained improvement at 1m	BFCRS (30)	20-30	Responded to ZM 20mg + ARP 15mg + MEM 20mg in 2 days (but no BZ)<br><br>BFCRS 30 to 11 in 4 days; and then 6 in 5 days (partial response)
2024	Wadhwa et al [20]<br>US	1	12 F	Psychosis with Catatonia, with decreasing food intake, "freezing" episodes, mutism, posturing, rigidity, incontinence	ANMDARE subacute onset<br>Dysautonomia<br>Hyperthermia<br><br>CSF positive for NMDAR Abs	5	RIS for 2 days, changed to OLZ; stopped after 4 days<br><br>LZM 1.75mg IV 4 hourly - no benefit<br><br>Plasmapheresis couldn't proceed<br>Methylprednisolone, IGs, prednisolone, rituximab<br><br>ECT - no sustained improvement	BFCRS (17)	7.5-25	ZM 7.5mg increased to 22.5mg after 1w, and then 25mg for 1m<br><br>BFCRS score 17 dropped to close to 0<br><br>Score 5 after 2m of ZM treatment<br><br>Partial response; weaned over several months

							LZM stopped due to "paradoxical reaction"<br><br>ZM tried next			
B1: Total cases = 8										
**B2. ZM as treatment with benzodiazepines[b]**										
2008	Coconcea [21] (Case 1) US	1	65 M	S-AD<br>Severe catatonia		2	Resistant to ECT and BZ<br><br>ZM tried next	None	30 (10 TDS)	Dramatic response to ZM; noted as partial response as details not available
2008	Coconcea [21] (Case 2) US	1	23 F	Paranoid SZ<br>Catatonia		2	Partial response to BZ<br><br>ZM tried next	None	Unclear	Marked improvement with ZM, relapsed when non-concordant with medication; noted as partial response as details not available
2015	Jaimes-Albornoz et al [22] (Case 1 of 2) Spain	1	65 F	Catatonia with staring, posturing, echo-phenomena, Automatic obedience, gegenhalten, ambitendency, perseveration	Fronto-temporal dementia; gastrostomy for food refusal	4	LZM 2.5mg + ZM 10mg	BFCRS (32)	10	Partially improved in 1w with LZM 2.5mg and ZM 10mg<br><br>Perseveration and mannerisms remained
2021	Mahgoub et al [23] US	1	26 M	BaD, past NMS with HPL, relapse of paranoia<br><br>Catatonia with mutism, staring, agitation, excitement, impulsivity, posturing, combativeness, grimacing, mannerisms	None	4	Trials of LZM 30mg/d, DZM 20mg BD and ZM 10mg were not successful<br><br>ECT and then clozapine 300mg/d and valproic acid 1000mg/d helpful	BFCRS (14)	10	No benefit with ZM – no details whether given separately or jointly with either LZM or DZM
2023	Bouaziz et al [24] France	1	70 F	SZ, catatonia with rigidity, mutism, waxy flexibility, posturing, negativism<br><br>Past h/o catatonia where ECT and not LZM was effective	Early NPH	4	Zuclopenthixol, LZM (7.5) and ZM (30) ineffective<br><br>10 ECTs led to remission but not possible to proceed with maintenance ECT during Covid, OLZ<br><br>ZM trial and LZM trial tried<br><br>Intensive tDCS 50 sessions helpful, with decrease in BFCRS score from 36 to 13, and then to 3 with 150 sessions	BFCRS (40)	30	No response to ZM 30mg and LZM 7.5mg trials; unclear whether together or not

2023	Greenstein et al [25]<br>US	1	65 F	OCD, Tourette's, anxiety<br><br>Malignant catatonia with rigidity, negativism, tachycardia, labile BP	Peritoneal adeno-CA, post-op delirium, and then acute cerebellar stroke (Infarct), query NMS, raised CK, multiple medical complications<br><br>Possible autoimmune encephalitis	4	Citalopram and clomipramine for years, LZM, HPL, quetiapine for agitation, dantrolene, AMD 400mg, IGs, methylprednisolone, valproic acid 1000mg/d; LZM 35mg/d (no effect)<br><br>ECT contraindicated due to stroke<br><br>ZM 10-30mg tried on day 39, along with LZM, AMD<br><br>Discharged on clomipramine 50mg BD, LZM 1mg TDS, ZM 10mg/d	None	10-30	Partial response to ZM 30mg + LZM (reduced to 8mg/d due to delirium) + AMD 400mg/d, and then plasmapheresis 5 days after starting ZM; remission on day 70 (31 days after starting ZM) ("no longer felt to be …catatonic")
2024	Veluri et al [26]<br>US	1	48 F	MDD, catatonia with decreased speech, stopped eating, reduced response to external stimuli, staring, incontinence<br><br>Past admission in Mexico	None	5	LZM 2mg IV QDS, Bupropion 150mg/d (later non-adherence), with improvement in 4 days; recurrence after tapering LZM to 1mg BD<br><br>ECT not an option; multiple ADs (not all specified)<br><br>Recurrence after stopping ZM few weeks later and then tapering LZM<br><br>Managed with LZM 5mg PO QDS, ZM 5mg BD, and MEM 20mg/d, Escitalopram 15mg/d (later changed to Mirtazapine 7.5mg/d)	BFCRS (14)	10 (5 BD)	ZM 5mg BD along with LZM 5mg IV QDS reduced BFCRS score from 14 to 6 (partial response)
2024	Midkiff et al [27]<br>Case 1 of 2<br>US	1	77 F	OCD, GAD, MDD, worse for 2m<br><br>Catatonia, with mutism, mitgehen, ambitendency, withdrawal, stupor, stereotypy, negativism, waxy flexibility, grasp reflex	Delirium	5	LZM 0.5mg IV resolution in 30min BFCRS score 20 to 15; LZM 0.5 BD resolved (BFCRS 1)<br><br>Developed delirium on Day 2 - LZM stopped, catatonia worsened with BFCRS score of 8; CZM 0.5mg BD - delirium worsened and catatonia worse with BFCRS score 21<br><br>ZM 5mg TDS tried	BFCRS (21)	15	Remission ("rapidly returned to her baseline state, with …BFCRS score of 0) in 24h to ZM 5mg TDS; BFCRS score 21 to 0<br><br>Delirium also improved; lack of clarity whether BZ were stopped.

B2: Total cases = 8										
Total cases (B1 + B2) = 16										
**C. ZM alone as both a challenge test and for treatment**										
1995	Mastain et al [28]<br>France<br><br>Additional details described in [6]	1	56 F	Catatonia with mutism, psychomotor slowing, verbal stereotypies, hypertonia, fixed gaze	None	7	Resistant to ECT and BZ (LZM and AZM)<br><br>No response to zopiclone<br><br>ZM 10mg tried	Unvalidated scale based on BFCRS Score (19/19)	10	Marked improvement (Partial response) with “double blind” challenge with ZM 10mg (catatonia score dropped from 19 to 14 on placebo, and 2 on ZM)<br><br>Similar effect seen next day on ZM rechallenge with remission<br><br>Remission for treatment (“all the signs disappeared under ZM”)
2013	Isomura et al [29]<br>Japan	1	69 F	Catatonia with little or no spontaneous movement, mutism, refusal to eat or drink	Frontotemporal dementia	5	No response to zopiclone, LZM, brotizolam, AZM, DZM, quazepam, quetiapine, fluvoxamine, lamotrigine, mirtazapine, and AMD<br><br>With ZM 5mg, perfusion was decreased in brainstem and improved in thalamus and some cortical areas, including left parieto-temporal lobe and left cerebellar cortex	BFCRS (26)	5	Response in 30 minutes; lasted for a few hours (BFCRS score dropped from 26 to 3, with only perseveration remaining) (partial response)<br><br>Effect sustained for 2y (remission) (”anti-catatonic signs of ZM …observed for these 2y”)
2013	Peglow et al [30]<br>US	1	46 M	S-AD<br>Catatonia with disorganised behaviour, mutism, and later, rigidity	Query NMS	6	Bupropion, RIS, lithium, low dose ARP– no response<br><br>6 unilateral ECTs: no effect Dantrolene, AMD, bromocriptine for atypical NMS, with improvement in NMS but not catatonia<br><br>Very agitated with LZM though rigidity improved<br><br>ZM tried alone<br><br>Discharged on ZM 10mg QDS + divalproex 1500mg/d	None	30-40	ZM test – improved rigidity and mutism (partial response)<br><br>5th day – ZM 10mg QDS<br><br>Remission (“complete resolution of his catatonia”) in 4w
2014	Appu & Noetzel [31]<br>US	1	16 F	Psychosis<br>Catatonia with agitation, perseveration, combativeness,	ANMDARE, left ovarian mature teratoma, MCS. CSF NMDA Abs positive	5	HPL, immunotherapy, steroids, tumour resection, plasmapheresis	None	10	Sudden improvement with ZM (for sleep), ZM weaned off 3w after discharge

				dyskinesias, autonomic instability, fever			ZM tried to improve sleep on Day 42<br><br>Almost recovered in 2m. Complete recovery in 6m			Marked as partial response for both challenge and treatment due to lack of detail regarding remission
2016	Amorim and McDade [32]<br>US	1	73 F	Catatonia with withdrawal, fluctuating mutism, reduced activity	Mature ovarian teratoma (ANMDAR Abs negative 10m post-resection), DM type II, atrial fibrillation, HT, hypertensive encephalopathy, Inclusion body myositis<br>Fluctuating confusion	6	12 ECTs (without any seizure elicitation) but partial response to later ECTs, tumour resection, BZ, APs, immunotherapy.<br>No response<br><br>ZM tried for sleep difficulties	None	5 (for sleep)	Quick response to ZM (exact gap not stated – "shortly after"); lasted for 6-10h<br><br>Later less (partial) response, with full effect lasting no more than 4-6h
2016	Rodgers and Arciniegas [10]<br>(Case 1 of 3)<br>US<br><br>Additional information obtained from the authors	1	Teenag e<br>F	Catatonia with immobility/stupor, staring, posturing, rigidity, grimacing, dysautonomia	Severe traumatic brain injury with MCS	4	AMD 200mg BD + Gabapentin for dysautonomia<br><br>ZM test	BFCRS (10)<br>CRS-R (12)	5-10	Temporary emergence from MCS (CRS-R score from 12 to 22) and reduced catatonia with both doses with partial response<br><br>Continued treatment with ZM helped improvement
2016	Bastiampillai et al [33]<br>Australia	1	59 F	BaD<br>Catatonia with mutism, echolalia, echopraxia, posturing	None	6	Past Lithium, carbamazepine, OLZ, amisulpride, periciazine, quetiapine<br><br>LZM 2mg TDS PO led to sedation and ataxia, with no improvement though BFCRS dropped from 18 to 12<br><br>24 bitemporal ECTs tried next: no response<br><br>ZM 5mg tried<br><br>No details about current medications except ZM 5mg followed by extended ECTs: led to complete resolution	BFCRS (18)	5	Partial response to ZM test in 15 minutes<br><br>Resolution (BFCRS from 18 to 4) in 1h and relapse in 5-6h.<br><br>Rechallenge – similar transient partial response

2019	Baptista and Choucha [34]<br>France	1	25 F	Psychosis, EPSEs with HPL<br>Catatonia with withdrawal, mutism, rigidity, immobility, waxy flexibility, catalepsy, posturing, staring, tremors, excitement, negativism	SLE with ANA Abs<br>AP-related catatonia	6	Methylprednisolone 500mg, then prednisolone 1mg/kg + hydroxy-chloroquine, cyclo-phosphamide, plasma exchange: no effect<br><br>HPL 5mg/d: AP-related catatonia<br><br>HPL stopped + LZM 4mg/d: resolution of catatonia, LZM stopped which worsened agitation<br><br>OLZ 20mg/d + high dose cyamemazine led to worsening of psychosis – changed to amisulpride + valproate 2g/d – no improvement<br><br>Loxapine 100mg IM + DZM 10mg IM single injection added – catatonic still signs and EPSE present<br><br>Tropatepine 10mg for EPSE: no effect<br><br>Amisulpride stopped and ZM tried – remission<br><br>Symptoms reappeared with loxapine for agitation but rechallenge with ZM, again achieved complete resolution of both catatonia and psychosis with BFCRS of 0. Valproate continued for mania.<br>DZM not helpful; ZM 10mg continued for >3y to prevent relapse	BFCRS (20)	10	Resolution of catatonia and psychosis with ZM in 30 minutes; lasted 3h.<br><br>Rechallenge with ZM, again achieved complete resolution of both catatonia and psychosis with BFCRS of 0.
2019	Ryan-Michailidis et al [35]<br>US	1	22 M	Catatonia with stupor, rigidity, posturing, mutism, stereotypies and dysautonomia	Cardiac arrest, status asthmaticus<br><br>Brain MRI: evidence of sequela of hypoxic ischemic injury including thalami	5	ZM 5mg single dose<br>No other details of treatment	BFCRS (28)<br>GCS (10)<br>DSM-5 catatonia	5-10	Response to ZM 5mg dose in 1h, with progressive improvement to ZM 10mg over days (BFCRS score dropped from 28 to 2)<br><br>Response lasting more than 3-4h

					swelling and corpus callosum infarct					
2020	Kumar and Kumar [36]<br>(Case 1 of 2)<br>India	1	15 F	Catatonia with background psychosis of 1m duration, with mutism, posturing, reduced oral intake, agitation, aggression, decreased social interaction	None	7	Favourable result with LZM challenge test.<br><br>Only transient response to LZM 2mg IV TDS over 2 days<br><br>Added OLZ 10mg/d for 3 days – no additional response<br><br>ECT couldn't be given to adolescents<br><br>ZM challenge test, followed by treatment	BFCRS (20)	10-30	Some improvement (partial response) in 30 minutes after ZM 10mg<br><br>Remission ("complete response in all the catatonic symptoms') in 3 days with ZM 30mg/d<br><br>Discharged on ZM 12.5mg TDS + OLZ 10mg/d<br><br>Follow up 2m later - remained well
2020	Kumar and Kumar [36]<br>(Case 2 of 2)<br>India	1	14 M	Catatonia of 20-day duration, with withdrawal, posturing, stopped eating/drinking, mutism, repetition of phrases, stiffness of body	None	6	Improvement on LZM challenge test (BFCRS score 22 at start; dropped to 18 with LZM). Then LZM 2mg IV TDS<br><br>OLZ 10mg/d (stopped due to akathisia), Propranolol up to 40mg/d<br><br>ZM challenge test, followed by treatment	BFCRS (22)	30-37.5	Improvement (partial response) in 30 minutes following ZM challenge test – no AEs<br><br>Discharged on ZM 12.5mg TDS<br><br>Remission at follow up 2m later - remained asymptomatic, after which ZM was tapered off and stopped
2023	Spiegel et al [37]<br>US	1	21 F	Psychosis<br>Catatonia, with stupor, mutism, staring	Left ovarian teratoma<br>ANMDARE (CSF positive for ANMDAR Abs) with psychosis and catatonia progressing to MCS<br><br>Orofacial dyskinesias<br>Dysautonomia	6	Tumour excision, IV IGs, plasmapheresis, IV dexmedetomidine: no response<br><br>ZM 5mg/d on Day 25 to 20mg/d on Day 28<br><br>Discharged to a rehab facility on ZM 5mg QDS on Day 50	BFCRS (23)<br>CRS-R (15)	5-20	MCS improved after a day and catatonia resolved after 8 days of ZM treatment (5mg/d on Day 25 to 5mg QDS after Day 28)<br><br>BFCRS dropped from 23 to 2 at discharge<br><br>CRS-R improved from 15 to 23 in 8 days though with residual cognitive (but not catatonic) symptoms
2024	Parasher et al [38]<br>India	1	20 M	Catatonic SZ, with mutism, wandering, posturing, violent behaviour, holding food in mouth for extended periods, poor self-care, staring	Large arachnoid cyst in the posterior fossa behind the cerebellar hemispheres with no mass effect	4	LZM 4mg IM: good response<br><br>OLZ 10mg + LZM 6mg + flupentixol 20mg IM depot - no change<br><br>LZM 16mg: BFCRS score dropped from 12 to 9	BFCRS (12)	30-40	Minimal response with ZM (BFCFRS score dropped from 9 to 8); recorded as non-response

							Added ZM 30mg: BFCRS score 8 from 9<br><br>Added ECTs: BFCRS score 0<br><br>Catatonic symptoms returned on stopping ECTs despite ZM 40mg/d and OLZ 20mg/d<br><br>Maintenance ECT weekly helpful			
2024	Yakubu et al [39]<br>UK	1	70 F	TRD (numerous ADs, lithium, anti-anxiety, and APs)<br>Catatonia, with mutism, withdrawal, agitation, excitement, grimacing, echolalia, stereotypies		5	No response to 15 ECTs, limited response to LZM 4-6mg/d; both stopped<br><br>ZM 10mg tried; increased to 10mg QDS with good improvement - so continued on the same dose along with Venlafaxine 112.5mg/d with marked improvement	BFCRS (17)	10-40	Partial response in 30 minutes to ZM 10mg (BFCRS score 17 to 6)
2024	Palma and Rayyan [40]<br>US	1	55 F	Past S-AD or BaD<br>Catatonia with fixed gaze, didn't follow commands or move extremities	CKD, acute hypoxic respiratory failure secondary to pneumonia, query vasculitis	5	Steroids, plasma exchange<br><br>Started on ZM 5mg TDS along with her "psychiatric medication"<br><br>Needed a tracheostomy	BFCRS (14)	10-15	Partial response to ZM 10mg in 30 minutes (BFCRS score 14 to 7)<br><br>Later ZM 5mg TDS
C1: Total cases = 15										
**C2. ZM as both a challenge test and treatment, with benzodiazepines[b]**										
2014	Hlal et al [41]<br>Morocco	1	24 F	First episode catatonia, with alternating stupor/ agitation, mutism, waxy flexibility, oppositional, posturing, echolalia, verbigeration<br><br>Catatonic SZ	NMS-like picture following OLZ 2.5mg/d	6	Worsening within 2 days after OLZ 2.5mg/d<br><br>DZM 40mg/d for 4w – no difference<br><br>ZM combined with DZM<br><br>Discharged on amisulpride 100mg/d	None	12.5-25	12.5mg – good and rapid (partial) response though didn't last for more than 2-4h; later increased to 12.5mg BD<br><br>Remission in 1m ("resolution of the catatonic picture, without reappearance of catatonia")
2015	Rajagopal [42]<br>India	1	71 F	Depressive episode<br>Catatonia with refusal to eat, mutism, posturing, psychomotor slowness,	Slightly raised TSH, MI 15y back, onset 1w after starting amiodarone	4	No antidepressant, amiodarone stopped<br><br>ZM 5mg/d + CZM 0.25mg TDS	None	5	Improvement (partial response) in functioning 1 day after ZM and CZM<br><br>Both stopped after 1w when she was in remission ("back to her normal self within the next 3w")

				uncooperative, staring			Digoxin for persistent atrial fibrillation			
2015	Javelot et al [43]<br>France	1	32 M	Paranoid SZ<br><br>3 episodes of catatonia (stupor and agitation)	None	5	1st Episode: OLZ 22.5mg/d + Imipramine<br><br>ZM 10mg TDS + tropatepine 20mg/d + LZM 1.5mg/d + OLZ 10mg/d<br><br>2nd Episode: ARP 15-30mg/d, later changed to RIS 4mg/d<br><br>LZM 6.25mg/d + tropatepine 30mg/d<br><br>ZM 30mg/d – partial response – then stopped after 2w – LZM 7.5mg/d – improved<br><br>3rd Episode (2y later despite LZM 7.5mg/d): amisulpride 300mg/d – later changed to OLZ 10mg/d<br><br>ZM 30mg/d added to LZM – good response; gradually discontinued over 10 days – no relapse of catatonia a year later	None	30	Good partial response to ZM and LZM<br><br>1st Episode: ZM stopped after 48h<br><br>Relapse of catatonia 2m after stopping ZM<br><br>3rd Episode: ZM 30mg/d led to good response
2019	Sayadnasiri and Rezvani [44]<br>Iran	1	65 F	Catatonia, with mutism, staring, sitting for long periods, resisted eating, uncooperative	Frontotemporal dementia<br>MRI: left frontal atrophy<br>MMSE 18<br>HT under control	6	LZM 8mg/d – BFCRS score dropped from 18 to 9 but still mute despite adding MEM 10mg/d<br><br>ZM 5mg added to LZM and MEM<br><br>Later ZM 10mg BD<br><br>LZM and ZM stopped due to sedation but no recurrence of catatonia after 2m	BFCRS (18)<br>Letter frequency test	5-20	Dramatic improvement in speech in 20 minutes after adding ZM to LZM and MEM; effect lasted for 2-3h<br><br>ZM 20mg + LZM + MEM – partial response
2023	Bonomo et al [45]<br>US	1	Middle age<br>F	PTSD with psychosis (previous diagnoses of BaD, SZ, psychotic depression), past sexual abuse, past cocaine misuse	Hypothyroidism<br>Rabbit syndrome	7	Lithium, SSRIs, lurasidone for presumed bipolar depression; prazosin for presumed PTSD<br><br>CZM 0.5mg BD (lack of response)	None	30 (10 TDS)	Response after even one dose of ZM (duration and dose not mentioned); was also on LZM initially at the time of challenge test<br><br>Then LZM stopped and ZM 10mg TDS continued for treatment in the absence of BZ

				Recurrent akinetic catatonia, with psychomotor slowing, mutism, waxy flexibility, stupor			Oral LZM 2mg TDS (lack of response and couldn't tolerate it)<br><br>Fludrocortisone (for hypotension with LZM)<br><br>Trial of oral ZM<br><br>Discharged on ZM 5mg TDS, lithium 600mg/d, lurasidone 40mg/d, tapered off in 1m and stayed well during follow-up of 6m			Catatonia remission ("her catatonia appeared fully broken") in few days to 10mg TDS ZM without AEs
C1: Total cases = 5										
C1 + C2: Total cases = 20										

Key: Abs: Antibodies; **AMD:** Amantadine; **ANMDAR**: Anti-NMDA receptor; **ANMDARE**: Anti-NMDA receptor encephalitis; **AD(s)**: Antidepressant(s); **AEs**: Adverse effects; **AF**: Atrial fibrillation; **ANA**: Antinuclear antibodies; **AP(s)**: Antipsychotic(s); **ARP**: Aripiprazole; **ASD**: Autism spectrum disorder; **AZM**: Alprazolam; **BaD**: Bipolar affective disorder; **BD**: twice a day; **BFCRS**: Bush-Francis catatonia rating scale; **BZ**: Benzodiazepine(s); **CA**: Carcinoma; **CABG**: Coronary artery bypass graft; **CAD**: Coronary artery disease; **CBZ**: Carbamazepine; **CK**: Creatine kinase; **CKD**: Chronic kidney disease; **CPZ**: Chlorpromazine; **CRF**: Chronic renal failure; **CSF**: Cerebrospinal fluid; **CRS-R**: Coma recovery scale – revised; **CZM**: Clonazepam; **d**: day(s); **DZM**: Diazepam; **DM**: Diabetes mellitus; **E**: Episode; **ED**: Eating disorder; **ECT**: Electroconvulsive therapy; **EEG**: Electroencephalography; **EPSE**: Extrapyramidal side-effects; **F**: Female; **GAD**: Generalised anxiety disorder; **GMC**: General medical conditions; **h**: hours; **H/o**: History of; **HPL**: Haloperidol; **HT**: Hypertension; **IGs**: Immunoglobulins; **IM**: Intramuscular; **IV**: Intravenous; **LZM**: Lorazepam; **OCD**: Obsessive compulsive disorder; **M**: Male; **MCS**: Minimally conscious state; **MDD**: Major depressive disorder; **MEM**: Memantine; **MI**: Myocardial infarction; **m**: month(s); **min**: minute(s); **MMSE**: Mini-mental state examination; **MRI**: Magnetic resonance imaging; **NA**: Not available; **NCRS**: Northoff catatonia rating scale; **NG**: Nasogastric tube; **NMS**: Neuroleptic malignant syndrome; **NPH**: Normal pressure hydrocephalus; **OLZ**: Olanzapine; **OZM**: Oxazepam; **PAHT**: Pulmonary artery hypertension; **PD**: Parkinson's disease; **PO**: Per oral; **PTSD**: Post-traumatic stress disorder; **QDS**: four times a day; **RIS**: Risperidone; **S-AD**: Schizoaffective disorder; **SLE**: Systemic lupus erythematosus; **SZ**: Schizophrenia/schizophreniform disorder; **tDCS**: transcranial Direct Current Stimulation; **TB**: Tuberculosis; **TDS**: three times a day; **TRD**: Treatment resistant depression; **TSH**: Thyroid stimulating hormone; **w**: week(s); **y**: year(s); **ZM**: Zolpidem

[a] Challenge test includes cases where there is a response noted to the first single dose of zolpidem
[b] Includes cases where zolpidem was used in a setting where benzodiazepines were also prescribed at the same time and timeline separating zolpidem and benzodiazepines wasn't clear.
[c] Oral zolpidem dose (mg)
[d] If a case described improvement but without clear details of a complete absence of catatonia with treatment, then a partial response was noted.

1. Rascle C, at al. Catatonia relief with zolpidem: an open study. *European Neuropsychopharmacology* 1997; 7: S277.
2. Thomas P, Rascle C, Mastain B, et al. Test for catatonia with zolpidem. *Lancet* 1997; 349: 702. DOI: 10.1016/S0140-6736(05)60139-0.
3. Zaw ZF and Bates GD. Replication of zolpidem test for catatonia in an adolescent. *Lancet* 1997; 349: 1914. DOI: 10.1016/s0140-6736(05)63915-3.
4. Zaw FK, Bates GD, Murali V, et al. Catatonia, autism, and ECT. *Dev Med Child Neurol* 1999; 41: 843-845. DOI: 10.1017/s001216229900167x.
5. Cottencin O, Warembourg F, de Chouly de Lenclave MB, et al. Catatonia and consultation-liaison psychiatry study of 12 cases. *Prog Neuropsychopharmacol Biol Psychiatry* 2007; 31: 1170-1176. 20070419. DOI: 10.1016/j.pnpbp.2007.04.006.
6. Thomas P, Cottencin O, Rascle C, et al. Catatonia in French psychiatry: implications of the zolpidem challenge test. *Psychiatric Annals* 2007; 37: 45.
7. Cottencin O, Danel T, Goudemand M, et al. Catatonia recognition and treatment. *Med Sci Monit* 2009; 15: CS129-131.
8. Bram D, Bubrovszky M, Durand JP, et al. Pernicious anemia presenting as catatonia: correlating vitamin B12 levels and catatonic symptoms. *Gen Hosp Psychiatry* 2015; 37: 273 e275-277. 20150223. DOI: 10.1016/j.genhosppsych.2015.02.003.
9. Ellul P, Rotge JY and Choucha W. Resistant Catatonia in a High-Functioning Autism Spectrum Disorder Patient Successfully Treated with Amantadine. *J Child Adolesc Psychopharmacol* 2015; 25: 726. 20150810. DOI: 10.1089/cap.2015.0064.
10. Rodgers J and Arciniegas D. Do catatonic features predict response to zolpidem in disorders of consciousness? *Brain Injury* 2016; 30(5-6): 695. Conference Abstract. DOI: https://dx.doi.org/10.3109/02699052.2016.1162060.
11. Seetharam P and Akerman RR. Postoperative echolalia and catatonia responsive to gamma aminobutyric acid receptor agonists in a liver transplant patient. *Anesth Analg* 2006; 103: 785-786. DOI: 10.1213/01.ANE.0000227163.12053.BE.
12. Almeida M, Cicolello K, Hanso A, et al. Treatment of Acute Agitation Associated With Excited Catatonia Using Dexmedetomidine: Case Series and Literature Review. *Prim Care Companion CNS Disord* 2021; 23 20211007. DOI: 10.4088/PCC.20cr02899.
13. Kocmur M and Vodopivec Z. Reappearance of catatonia. A case report. *Neuro Endocrinol Lett* 2006; 27: 260-262.
14. Bation R, Devic P, Lambrinidis A, et al. Recurrent Self-Limited Hyperthermia Following ECT for Catatonia in a Young Man with Cerebral Palsy. *Psychosomatics* 2012; 53(5): 474-477. DOI: https://dx.doi.org/10.1016/j.psym.2011.08.013.
15. Kapulsky L, Greiner MG, Daniels JE, et al. Clozapine Discontinuation and Malignant Catatonia: A Case Report. *Psychosomatics* 2019; 60: 75-79. 20180321. DOI: 10.1016/j.psym.2018.03.007.
16. Yamaguchi H, Chiba Y, Katsuse O, et al. A case of subclinical Cushing's syndrome presenting as catatonia. *Psychogeriatrics* 2019; 19(4): 402-403. Note. DOI: https://dx.doi.org/10.1111/psyg.12394.
17. Bean E, Findlay C, Gee C, et al. Refractory catatonia in old age: a case report. *J Med Case Rep* 2021; 15: 406. 20210814. DOI: 10.1186/s13256-021-03000-3.
18. Wendt L, Hornung M and Sami R. Zolpidem in Elderly Patients With Acute Treatment Resistant Catatonia: A Case Report. *Hosp Pharm* 2023; 58: 444-447. 20230322. DOI: 10.1177/00185787231161514.
19. Cruzado L and Hurtado-Llamo ND. Apparent rapid response to the combination of zolpidem, memantine and aripiprazole in a case of schizophrenic catatonia. [Spanish]. *Revista de Neuro-Psiquiatria* 2023; 86(3): 245-250. DOI: https://dx.doi.org/10.20453/rnp.v86i3-1.4980.
20. Wadhwa A, Vidal C, Saade Y, et al. Alternative treatment in adolescent with anti-NMDA receptor encephalitis catatonia. *Neurocase* 2024; 30: 234-237. 20241204. DOI: 10.1080/13554794.2024.2436214.

21. Coconcea C. Zolpidem in treatment resistant catatonia: 2 case reports and literature review. *Schizophrenia Research* 2008; 98: 1.
22. Jaimes-Albornoz W, Ballesteros-Prado A and Serra-Mestres J. Catatonia in patients with frontotemporal dementia. *European Psychiatry* 2015; 1): 1436. Conference Abstract.
23. Mahgoub Y, Makar M and Virani S. When Nothing Stops Catatonia Except Electroconvulsive Therapy and Maybe A New Savior, Clozapine. *Primary Care Companion for CNS Disorders* 2021; 23(4) (no pagination). DOI: https://dx.doi.org/10.4088/PCC.20l02823.
24. Bouaziz N, Luisada JC, Jabri S, et al. Moving to accelerated protocols of tDCS in catatonia: a case report. *Frontiers in Psychiatry* 2023; 14(no pagination). DOI: https://dx.doi.org/10.3389/fpsyt.2023.1302718.
25. Greenstein SP, Petrides G and Fricchione G. Consultation-Liaison Case Conference: Malignantly Catatonic and Unable to Receive Electroconvulsive Therapy. *Journal of the Academy of Consultation-Liaison Psychiatry* 2023; 64(2): 158-165. Review. DOI: https://dx.doi.org/10.1016/j.jaclp.2022.10.262.
26. Veluri N, Wise W, Kutcher R, et al. The multi-modal treatment of catatonia: Targeting multiple receptors when GABA-based benzodiazepines fail. *Personalized Medicine in Psychiatry* 2024; 43-44(no pagination). DOI: https://dx.doi.org/10.1016/j.pmip.2024.100121.
27. Midkiff JP, Gaba A and Munjal S. When Lorazepam Worsens Delirium in Your Catatonic Patient. *Primary Care Companion for CNS Disorders* 2024; 26(1) (no pagination). Note. DOI: https://dx.doi.org/10.4088/PCC.23cr03616.
28. Mastain B, Vaiva G, Guerouaou D, et al. [Favourable effect of zolpidem on catatonia]. *Rev Neurol (Paris)* 1995; 151: 52-56.
29. Isomura S, Monji A, Sasaki K, et al. FTD with catatonia-like signs that temporarily resolved with zolpidem. *Neurol Clin Pract* 2013; 3: 354-357. DOI: 10.1212/CPJ.0b013e318296f263.
30. Peglow S, Prem V and McDaniel W. Treatment of catatonia with zolpidem. *J Neuropsychiatry Clin Neurosci* 2013; 25: E13. DOI: 10.1176/appi.neuropsych.11120367.
31. Appu M and Noetzel M. Clinically significant response to zolpidem in disorders of consciousness secondary to anti-N-methyl-D-aspartate receptor encephalitis in a teenager: a case report. *Pediatr Neurol* 2014; 50: 262-264. 20131113. DOI: 10.1016/j.pediatrneurol.2013.11.001.
32. Amorim E and McDade EM. Rapidly-progressive catatonia responsive to zolpidem in a patient with ovarian teratoma-associated paraneoplastic encephalitis. *J Clin Neurosci* 2016; 30: 136-138. 20160307. DOI: 10.1016/j.jocn.2016.01.028.
33. Bastiampillai T, McGovern V, Lloyd B, et al. Treatment refractory chronic catatonia responsive to zolpidem challenge. *Aust N Z J Psychiatry* 2016; 50: 98. 20150423. DOI: 10.1177/0004867415582232.
34. Baptista A and Choucha W. Dramatic and Rapid Resolution of Both Psychosis and Neuroleptic-Related Catatonia With Zolpidem in a Patient With Systemic Lupus Erythematosus. *J Clin Psychopharmacol* 2019; 39: 509-511. DOI: 10.1097/JCP.0000000000001088.
35. Ryan-Michailidis E, Badri M, Horng J, et al. Certain catatonic features in disorders of consciousness may predict response to zolpidem: A case report in an anoxic brain injury patient. *PM and R* 2019; 11(Supplement 2): S77. Conference Abstract. DOI: https://dx.doi.org/10.1002/pmrj.12271.
36. Kumar P and Kumar D. Zolpidem in treatment resistant adolescent catatonia: a case series. *Scand J Child Adolesc Psychiatr Psychol* 2020; 8: 135-138. 20200915. DOI: 10.21307/sjcapp-2020-013.
37. Spiegel DR, Ahuja K, Reid FE, et al. A Case of Ovarian Teratoma-Induced N-Methyl-D-Aspartate Receptor Antibody Encephalitis Presenting With Psychosis and Catatonia, Progressing to the Minimally Conscious State and Successfully Treated With Adjunctive Zolpidem. *Prim Care Companion CNS Disord* 2023; 25 20230801. DOI: 10.4088/PCC.22cr03466.
38. Parasher G, Yadav BK and Kar SK. Electroconvulsive therapy: a last resort for lorazepam-resistant catatonia in patient with large arachnoid cyst. *CNS Spectr* 2024; 29: 83-84. 20231020. DOI: 10.1017/S109285292300634X.

39. Yakubu AO, Gibson S and Smit R. The Use of Zolpidem in Catatonia: A Case Report. *Cureus* 2024; 16: e73493. 20241111. DOI: 10.7759/cureus.73493.
40. Palma CF and Rayyan E. A Case of Catatonia in the ICU. *American Journal of Respiratory and Critical Care Medicine Conference: American Thoracic Society* 2024; 209. Conference Abstract.
41. Hlal H, Kettani N, Berhili N, et al. [The role of zolpidem in improving catatonic schizophrenia. Case report]. *Presse Med* 2014; 43: 1018-1020. 20140413. DOI: 10.1016/j.lpm.2013.11.023.
42. Rajagopal S. Catatonic depression precipitated by amiodarone prescribed for atrial fibrillation. *Indian Journal of Psychiatry* 2015; 57(1): 105-106. Letter. DOI: https://dx.doi.org/10.4103/0019-5545.148545.
43. Javelot H, Michel B, Steiner R, et al. Zolpidem test and catatonia. *J Clin Pharm Ther* 2015; 40: 699-701. 20151107. DOI: 10.1111/jcpt.12330.
44. Sayadnasiri M and Rezvani F. Treatment of Catatonia in Frontotemporal Dementia: A Lesson From Zolpidem Test. *Clin Neuropharmacol* 2019; 42: 186-187. DOI: 10.1097/WNF.0000000000000362.
45. Bonomo N, Huang H and Schoenbachler B. Rapid resolution of catatonia secondary to post traumatic stress disorder with secondary psychotic features through scheduled zolpidem tartrate. *BMC Psychiatry* 2023; 23: 258. 20230417. DOI: 10.1186/s12888-023-04769-x.
